# Prior dengue virus infection and risk of Zika: A pediatric cohort in Nicaragua

**DOI:** 10.1371/journal.pmed.1002726

**Published:** 2019-01-22

**Authors:** Aubree Gordon, Lionel Gresh, Sergio Ojeda, Leah C. Katzelnick, Nery Sanchez, Juan Carlos Mercado, Gerardo Chowell, Brenda Lopez, Douglas Elizondo, Josefina Coloma, Raquel Burger-Calderon, Guillermina Kuan, Angel Balmaseda, Eva Harris

**Affiliations:** 1 Department of Epidemiology, School of Public Health, University of Michigan, Ann Arbor, Michigan, United States of America; 2 Sustainable Sciences Institute, Managua, Nicaragua; 3 Division of Infectious Diseases and Vaccinology, School of Public Health, University of California, Berkeley, Berkeley, California, United States of America; 4 Laboratorio Nacional de Virología, Centro Nacional de Diagnóstico y Referencia, Ministry of Health, Managua, Nicaragua; 5 Georgia State University, Atlanta, Georgia, United States of America; 6 Health Center Sócrates Flores Vivas, Ministry of Health, Managua, Nicaragua; Mahidol-Oxford Tropical Medicine Research Unit, THAILAND

## Abstract

**Background:**

Zika virus (ZIKV) emerged in northeast Brazil in 2015 and spread rapidly across the Americas, in populations that have been largely exposed to dengue virus (DENV). The impact of prior DENV infection on ZIKV infection outcome remains unclear. To study this potential impact, we analyzed the large 2016 Zika epidemic in Managua, Nicaragua, in a pediatric cohort with well-characterized DENV infection histories.

**Methods and findings:**

Symptomatic ZIKV infections (Zika cases) were identified by real-time reverse transcription PCR and serology in a community-based cohort study that follows approximately 3,700 children aged 2–14 years old. Annual blood samples were used to identify clinically inapparent ZIKV infections using a novel, well-characterized serological assay. Multivariable Poisson regression was used to examine the relation between prior DENV infection and incidence of symptomatic and inapparent ZIKV infection. The generalized-growth method was used to estimate the effective reproduction number. From January 1, 2016, to February 28, 2017, 560 symptomatic ZIKV infections and 1,356 total ZIKV infections (symptomatic and inapparent) were identified, for an overall incidence of 14.0 symptomatic infections (95% CI: 12.9, 15.2) and 36.5 total infections (95% CI: 34.7, 38.6) per 100 person-years. Effective reproduction number estimates ranged from 3.3 to 3.4, depending on the ascending wave period. Incidence of symptomatic and total ZIKV infections was higher in females and older children. Analysis of the effect of prior DENV infection was performed on 3,027 participants with documented DENV infection histories, of which 743 (24.5%) had experienced at least 1 prior DENV infection during cohort follow-up. Prior DENV infection was inversely associated with risk of symptomatic ZIKV infection in the total cohort population (incidence rate ratio [IRR]: 0.63; 95% CI: 0.48, 0.81; *p <* 0.005) and with risk of symptomatic presentation given ZIKV infection (IRR: 0.62; 95% CI: 0.44, 0.86) when adjusted for age, sex, and recent DENV infection (1–2 years before ZIKV infection). Recent DENV infection was significantly associated with decreased risk of symptomatic ZIKV infection when adjusted for age and sex, but not when adjusted for prior DENV infection. Prior or recent DENV infection did not affect the rate of total ZIKV infections. Our findings are limited to a pediatric population and constrained by the epidemiology of the site.

**Conclusions:**

These findings support that prior DENV infection may protect individuals from symptomatic Zika. More research is needed to address the possible immunological mechanism(s) of cross-protection between ZIKV and DENV and whether DENV immunity also modulates other ZIKV infection outcomes such as neurological or congenital syndromes.

## Abstract 2

Eva Harris and colleagues reveal the protective effect of previous dengue virus infection against symptomatic Zika in a large pediatric cohort in Nicaragua during the 2016 Zika epidemic

## Author summary

**Why was this study done?:**

- Zika virus (ZIKV) was introduced into the Americas—a region with high levels of dengue virus (DENV) immunity—in 2015.
- Immunity generated by infection with one DENV serotype impacts the outcomes of subsequent infection with a different DENV serotype.
- Because ZIKV and DENV are closely related flaviviruses, it is possible that pre-existing DENV immunity may also impact susceptibility to Zika.

**What did the researchers do and find?:**

- In 2016, we followed the introduction and spread of ZIKV in a large, long-term cohort of children in Managua, Nicaragua, to determine who was infected with ZIKV and, of those, who developed symptomatic infection. For most of these children, the history of prior DENV infection had been well characterized.
- We found that children with prior DENV infection had lower rates of symptomatic Zika than children who did not have prior DENV infection.
- However, we found that prior DENV immunity did not affect the overall rate of ZIKV infection in children.

**What do these findings mean?:**

- These findings support the idea that prior DENV immunity might cross-protect against symptomatic Zika.
- Future studies are required to address the immunological mechanisms of cross-protection observed, as well as to examine whether prior DENV immunity impacts severe ZIKV infection outcomes such as neurological or congenital syndromes.

## Introduction

Zika virus (ZIKV) belongs to the *Flavivirus* genus in the *Flaviviridae* family. It was initially isolated in Uganda in 1947 [1], and the first evidence of human ZIKV infection came from serological studies conducted in Uganda in 1952 [2]. While the first human case was described in 1964 [3], few cases were detected until 2007, though serological evidence existed of circulation in Africa and southeast Asia [4]. In 2007, the first major outbreak of Zika occurred on Yap Island [5,6], and larger outbreaks were documented in Oceania in 2013–2014, in particular in French Polynesia [7]. ZIKV caused a major epidemic in Brazil starting in 2015, which rapidly spread to South, Central, and North America and the Caribbean. In Nicaragua, the first Zika case was identified in January 2016.

Acute ZIKV infection is often asymptomatic, and Zika was initially described as a mild infection, with the most commonly reported symptoms being rash, low-grade fever, arthralgia, and conjunctivitis [5]. However, during the French Polynesia outbreak, an increase in cases of Guillain–Barré syndrome (GBS) was reported, and retrospective studies led to the association of ZIKV infection with this neurological syndrome [8,9]. The association of ZIKV with GBS was also observed in the Americas in 2015–2016 [10]. Moreover, in the American pandemic, ZIKV infection during pregnancy has been shown to cause multiple congenital defects, most notably microcephaly [11,12].

ZIKV shares extensive homology with dengue virus (DENV). The 4 DENV serotypes interact immunologically: infection with one serotype provides transient cross-protection against infection with heterologous serotypes, but sequential infection with different DENV serotypes is the most important risk factor for severe dengue disease, an effect that is mediated in part by antibody-dependent enhancement [13–15]. In in vitro and murine models, both cross-neutralization and enhancement between DENV and ZIKV have been observed [16–19]. However, experiments to date in rhesus macaques [20] and viral load and cytokine analysis in humans [21] do not support ZIKV enhancement by preexisting DENV immunity. Interestingly, throughout the Americas, a precipitous decrease in the number of dengue cases was observed following widespread Zika epidemics [22], suggesting that ZIKV infection might induce cross-protective immune responses against DENV. However, the characterization of potential cross-protection between DENV and ZIKV requires knowledge of longitudinal pre-infection immune histories, which is only available in prospective cohort studies [23].

Here, we describe the introduction of ZIKV into the Pediatric Dengue Cohort Study (PDCS), a long-standing pediatric dengue cohort established in 2004 in Managua, Nicaragua, in which the DENV immune history of the participants is well-characterized [24–26]. The incidence of symptomatic and inapparent ZIKV infections from January 1, 2016, to February 28, 2017, was estimated, together with associated demographic risk factors. The effect of prior DENV infection on ZIKV infection and disease was also analyzed.

## Methods

### Ethics statement

The PDCS was reviewed and approved by the institutional review boards of the University of California, Berkeley (protocol 2010-09-2245; S1 and S2 Appendices), the University of Michigan (study ID: HUM00091606), and the Nicaraguan Ministry of Health (protocol NIC-MINSA/CNDR CIRE-09/03/07-008). Parents or legal guardians of all participants provided written informed consent, and participants 6 years of age and older provided oral assent. The protocol was amended in July 2015 to include screening for ZIKV infection in participants meeting the study testing definition and again in February 2016 to expand the testing definition (see below).

### Study population

The PDCS is an ongoing study of dengue (since August 2004), chikungunya (since September 2014), and Zika (since July 2015). Study design, population, and detailed methods have been described previously [25–27]. Briefly, the PDCS is a community-based prospective study consisting of approximately 3,700 children uniformly distributed over each year of age between 2 and 14 years. The study was sized to examine the effects of repeat DENV infection. The study is based at a primary health center, the Health Center Sócrates Flores Vivas (HCSFV), in District II of Managua, the capital of Nicaragua. The study area consists of 17 neighborhoods, with most inhabitants living at low to middle socioeconomic status. Primary healthcare is provided by study personnel to all participants, and acute illnesses are screened using the study testing definition (see below). Initial recruitment into the study occurred through door-to-door visits. All children 2 to 9 years old living within the study area were invited to participate; the age range was then extended to 14 years old. Every March, healthy blood samples (annual samples) are collected, and additional participants are enrolled to maintain the cohort age structure and compensate for loss to follow-up. Children aged 2 years are also enrolled year-round to maintain the age structure.

### Testing definition and laboratory assays

Acute (at presentation, day 1–5 after onset of symptoms) and convalescent (day 14–21 after onset of symptoms) blood samples were collected from participants presenting to the HCSFV with (1) fever or feverishness with 2 or more of the following symptoms: headache, muscle ache, joint pain, retro-orbital pain, rash, hemorrhagic manifestations, or leukopenia (1975–1997 WHO dengue case definition [28,29]); (2) fever or feverishness with 2 or more of the following: nausea/vomiting, rash, aches and pains, positive tourniquet test, leukopenia, or any warning sign (2009 WHO dengue case definition [30]); (3) undifferentiated fever; or (4) rash, regardless of additional signs and symptoms. The fourth category was included starting February 2016. A urine sample was collected from participants meeting these testing definitions and presenting Monday to Friday, 7 AM to 5 PM. RNA was extracted from acute serum and, as available, urine samples (QIAamp Viral RNA Mini Kit, Qiagen) and tested by real-time reverse transcription PCR (rRT-PCR) for ZIKV, DENV, and chikungunya virus RNA. Testing was performed in multiplex for the 3 viral RNAs [31,32] or separately using a singleplex ZIKV assay [6] and a multiplex DENV/chikungunya virus assay [33]. Paired acute and convalescent samples were tested using in-house ZIKV and DENV IgM antibody capture ELISAs (MAC-ELISA) and ZIKV and DENV inhibition ELISAs (iELISA) [15,26,34]. Additionally, pre- and post-infection (i.e., 2016 and 2017) annual samples were analyzed using a ZIKV NS1 blockade-of-binding (BOB) ELISA [34,35]. An algorithm was developed to integrate qualitative results from the 2 MAC-ELISAs and the ZIKV NS1 BOB assay, as well as quantitative results from the iELISAs (see below). Illness episodes meeting the testing definition were considered Zika cases (i.e., symptomatic ZIKV infections) if (1) ZIKV RNA was detected by rRT-PCR in serum and/or urine and/or (2) serological test results were consistent with a ZIKV infection using the algorithm developed. Participants whose paired annual samples (i.e., 2016 and 2017) showed a ZIKV NS1 BOB ELISA seroconversion but who were not identified as a Zika case were considered to have experienced an inapparent ZIKV infection. Total ZIKV infections refers to all identified ZIKV infections, whether a Zika case or an inapparent ZIKV infection.

### Classification algorithm

Classification trees (rpart package in R [36,37]) were used to implement a method called recursive partitioning to group cases by class based on shared characteristics in predictor variables. The classification tree was trained with rRT-PCR-confirmed Zika cases (*n =* 368; 3 excluded because of ZIKV/DENV co-infections and 3 did not have sufficient serological data to be used in the training set), rRT-PCR-confirmed dengue cases that occurred before (*n =* 97) or during (*n =* 13) the introduction of ZIKV, other febrile illnesses (OFIs) that occurred before the Zika epidemic (*n =* 75), and OFIs that occurred during the study period in children with rRT-PCR-confirmed ZIKV infection (*n =* 150). For all cases, we measured antibodies in acute and convalescent serum samples with the DENV and the ZIKV iELISAs as well as DENV and ZIKV MAC-ELISAs [25,34]. The annual sample after each case was tested using the ZIKV NS1 BOB ELISA [35].

To build the classification tree, each variable was tested for the cutoff value that best split the cases into their true categories. The variable that performed best became the top “rule” in the tree, splitting cases into 2 groups. The same procedure was repeated for each group separately, with the variable that best separated the groups into true classes chosen as the subsequent rule. This process of splitting continued until each case was correctly classified, producing a tree that was over-fit to the training dataset. To identify a tree for categorization of the datasets, we pruned the tree to the smallest tree for which misclassification was minimized in cross-validation. This subtree had the lowest complexity parameter, meaning the lowest misclassification of cases in *n*-fold cross-validation experiments, where 1 case is excluded when creating the tree and the resulting tree is used to estimate the class of the excluded case. We then selected the simplest tree (fewest “rules”) that had a complexity value within 1 standard error of the lowest complexity parameter tree. Optimal splits in the tree were identified using the Gini index, an impurity function. We included cases with missing data when creating the algorithm and allowed surrogate variables, i.e., variables that approximate the categorization achieved by the best splitting variable, to inform splits to enable classification of cases with missing data. The final algorithm was applied to classify all 1,111 cases that occurred during the study period. For cases that were rRT-PCR-positive for ZIKV or DENV, rRT-PCR was used to define the case rather than the algorithm.

### DENV infections and prior immunity

Symptomatic and inapparent DENV infections have been recorded in the PDCS since study inception in August 2004 through a combination of rRT-PCR, virus isolation, and serological methods for symptomatic cases, and dengue iELISA on paired annual samples for inapparent infections [25,26]. Infecting DENV serotype information is available for most symptomatic cases but only for a subset of the inapparent infections [38]. Prior DENV infection was defined as at least 1 inapparent or symptomatic infection since the participant entered the PDCS until the 2015/2016 season (from March 1, 2015, to February 29, 2016). Recent DENV infection was defined as an inapparent or symptomatic infection during the 2015/2016 season. Children with a documented prior DENV infection or who entered the cohort DENV-naïve and had no documented DENV infections were considered to have known DENV infection histories. Among children with a documented prior DENV infection, those who entered the cohort DENV-naïve and had a single documented DENV infection were considered as having primary DENV immunity, while those who entered the cohort DENV-immune and experienced one or more DENV infections and those who entered the cohort DENV-naïve and had two or more DENV infections were considered as having secondary DENV immunity.

### Statistical analysis

Data were analyzed using Stata v14 (StataCorp, College Station, TX). Relative and absolute frequencies are reported for categorical variables, and mean and standard deviation are reported for quantitative variables. Follow-up time was calculated as the amount of time between January 1, 2016, or enrollment, whichever came later, and February 28, 2017. For those lost to follow-up, follow-up was calculated as one-half the amount of time between last contact with study personnel and the date recorded as lost to follow-up. A Poisson distribution was used to calculate 95% confidence intervals (CIs) for incidence rates. A binomial distribution was used to calculate 95% CIs for seroprevalence and proportion of cases among infections. We used generalized estimating equations assuming a Poisson distribution to calculate incidence rate ratios (IRRs) for the risk of symptomatic ZIKV infection. Crude and adjusted IRRs were calculated using univariate and multivariate analysis, respectively. For analyses examining the risk of ZIKV infection and the risk of symptomatic presentation among all ZIKV infections, Poisson regression with robust standard errors was used. Analyses of the effect of DENV immunity were limited to children with documented DENV infection histories. In addition, all ZIKV cases or infections that occurred prior to March 1, 2016 (*n =* 68) were excluded because (1) the testing definition for Zika changed in February 2016 and (2) there is potentially DENV/ZIKV serological cross-reactivity in the ZIKV NS1 BOB assay when performed in samples collected early after the acute infection. Further, analysis of the effect of prior DENV immunity on Zika cases among ZIKV infections was limited to individuals who demonstrated a ZIKV NS1 BOB seroconversion, regardless of case status. Age was explored as a categorical and linear variable, and although the variable form did not impact the conclusions of the models, the continuous version generated a better model fit and thus was included in the final models.

### Characterizing reproduction number and early transmission phase

To estimate the effective reproduction number (*R*_e_) during the early phase of the Zika epidemic in Managua, we employed the generalized-growth method (GGM) [39], which links the generation interval of the disease [40] with case incidence series to extract transmission potential over time [39]. The power of the GGM lies in its flexibility to produce a range of growth dynamics values via 2 parameters: the growth rate (*r*) and the epidemic growth scaling (*p*). This growth dynamics value ranges from constant incidence (*p =* 0) to exponential growth (*p =* 1) [39].

## Results

### Study population

A total of 3,893 children aged 2–14 years participated in the PDCS between January 1, 2016, and February 28, 2017. Of these, 3,053 (78.4%) participated throughout the entire study period; 386 (9.9%) were enrolled after January 1, 2016; 440 (11.3%) withdrew or were withdrawn from the study; and 14 (0.4%) both enrolled after January 1, 2016, and withdrew. A majority of the individuals who did not participate in the entire study period were 2-year-old children who were enrolled after January 1, 2016, or children who turned 15 years and aged out of the cohort during the follow-up period. Other than the 15-year-old individuals who aged out of the cohort, children who were withdrawn from the cohort or lost to follow-up were demographically similar to the cohort population. Participants were uniformly distributed by sex and year of age (Table 1).

**Table 1:** Participant characteristics, Managua, Nicaragua, January 2016–February 2017.

Characteristic	Full cohort *n =* 3,893	Zika cases *n =* 560	Non-cases *n* = 3,333
**By sex**			
Female	1,944 (49.9)	309 (55.2)	1,635 (49.0)
Male	1,949 (50.1)	251 (44.8)	1,698 (51.0)
**By age (years)**			
2–5	1,146 (29.4)	128 (22.9)	1,018 (30.5)
6–9	1,249 (32.1)	205 (36.6)	1,044 (31.3)
10–14	1,498 (38.5)	227 (40.5)	1,271 (38.1)

Data presented as *n* (percent).

### Zika cases in the PDCS

**Fig 1:**
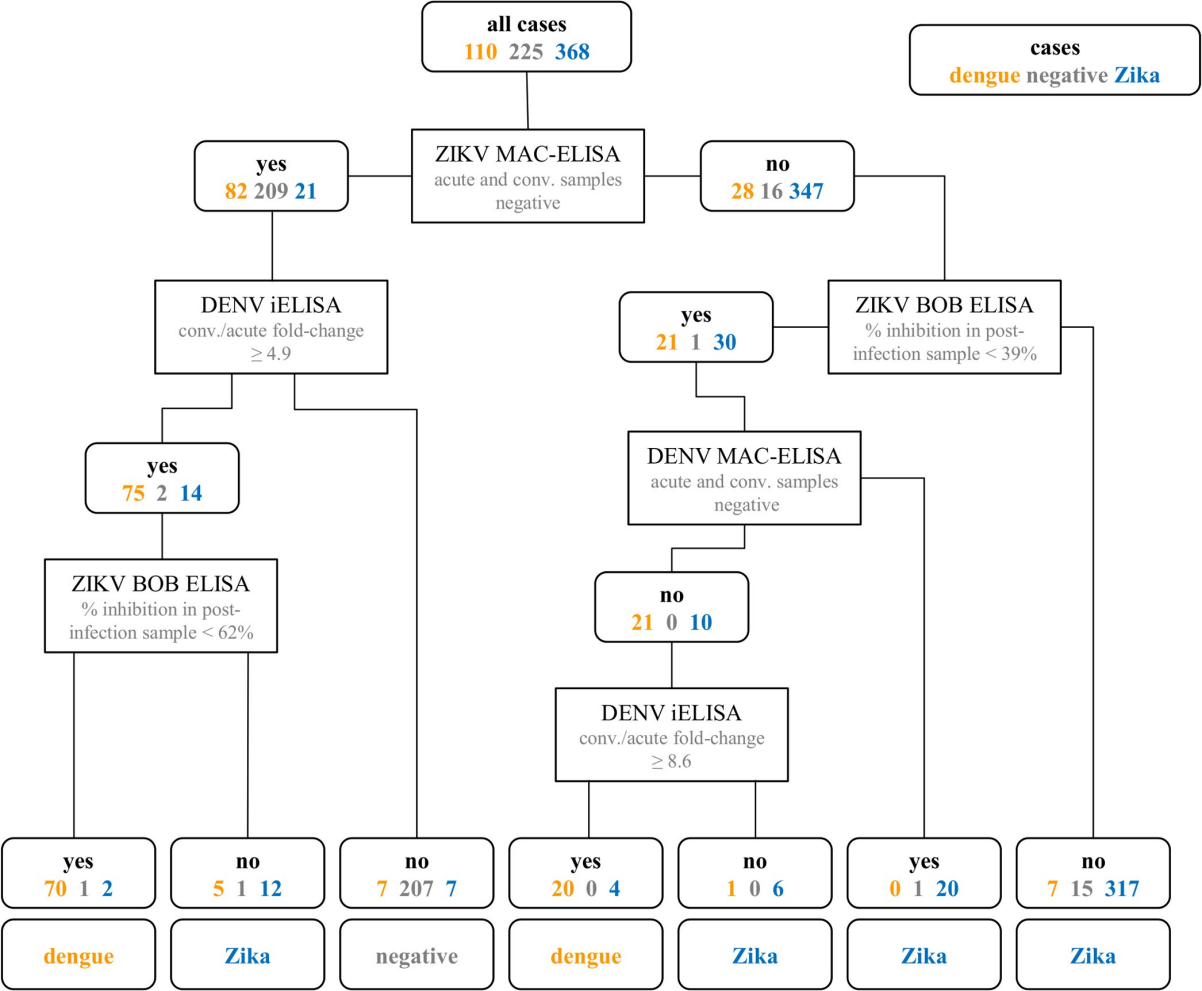
Classification tree showing the algorithm for distinguishing symptomatic Zika cases, dengue cases, and non-cases (negative cases) based on serological data. Results from 5 serological assays (ZIKV and DENV MAC-ELISAs on acute and convalescent samples, ZIKV and DENV iELISAs on acute and convalescent samples, and ZIKV NS1 BOB ELISA on the post-infection annual sample) were used to classify the cases. The numbers of rRT-PCR-confirmed Zika, dengue, and negative cases according to their assay results are shown in blue, orange, and grey, respectively. BOB, blockade-of-binding; conv., convalescent; DENV, dengue virus; iELISA, inhibition ELISA; MAC-ELISA, IgM antibody capture ELISA; rRT-PCR, real-time reverse transcription PCR; ZIKV, Zika virus.

Over the study period, 946 children presented 1,111 times with symptoms meeting the study testing definition. A total of 374 cases were positive for ZIKV by rRT-PCR. As many symptomatic ZIKV infections might present clinically after the detectable viremic phase of disease, we also developed an algorithm for serological diagnosis of Zika using the classification tree method (see methods above; Fig 1). The sensitivity and specificity was 96% and 91%, respectively, for ZIKV rRT-PCR-positive cases; 92% and 97% for OFI cases; and 82% and 99% for DENV rRT-PCR-positive cases. We applied the algorithm produced by the classification tree to the 1,111 cases of illness meeting the testing definition that occurred during the study period. Taking into account both the ZIKV rRT-PCR results and the algorithm, a total of 560 children were laboratory-confirmed as symptomatic ZIKV infections (Zika cases). Of the 560 Zika cases, 354 (63.2%) were confirmed by both rRT-PCR and the serology algorithm. Additionally, 186 cases (33.2%) were confirmed by serology only and 20 cases (3.6%) by rRT-PCR only.

**Fig 2:**
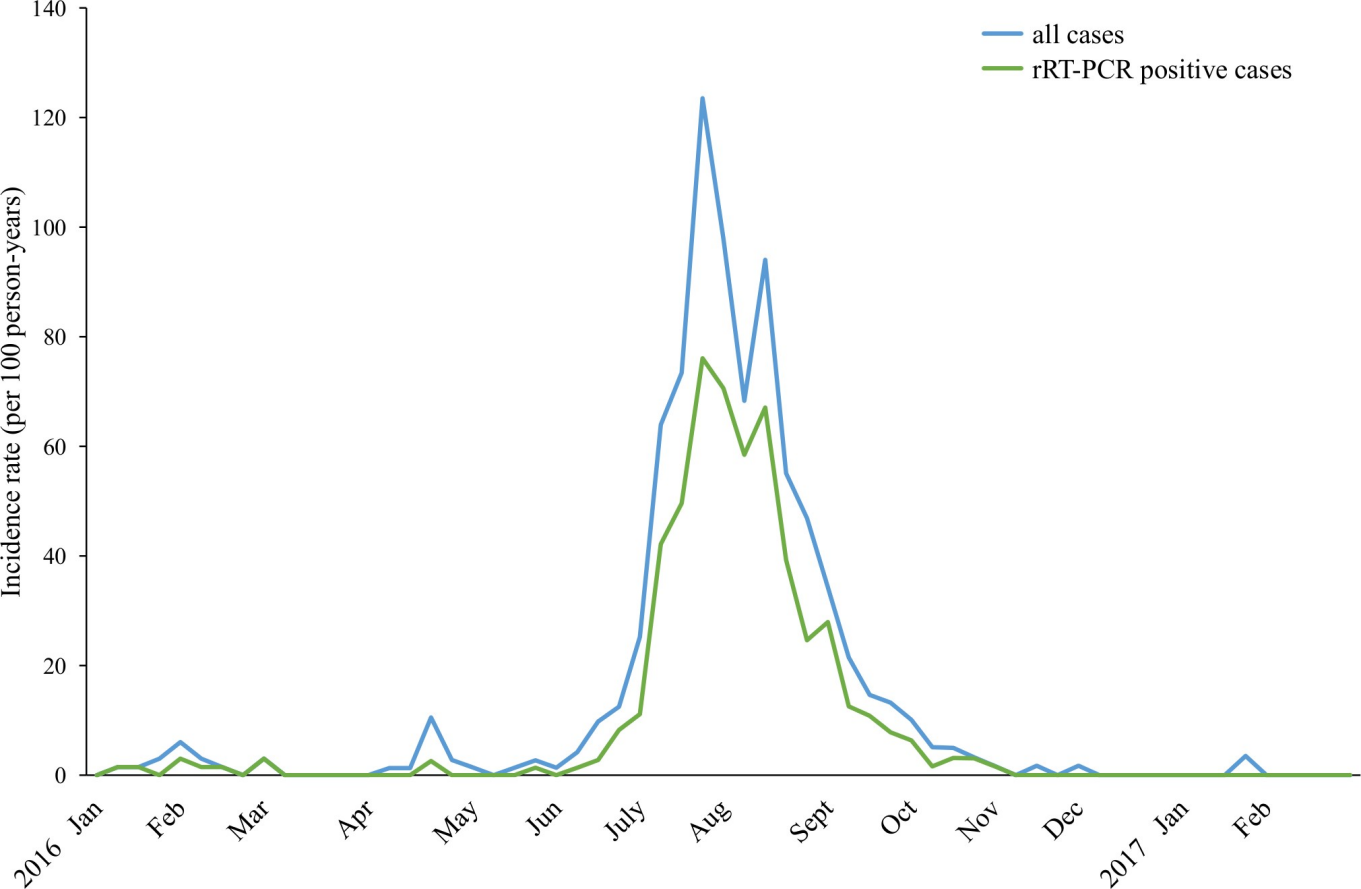
Weekly incidence of Zika in the Pediatric Dengue Cohort Study, January 2016–February 2017. Incidence of symptomatic ZIKV infection (all cases detected by rRT-PCR and/or the serological algorithm) and incidence of rRT-PCR-positive cases. rRT-PCR, real-time reverse transcription PCR; ZIKV, Zika virus.

The first Zika case in the cohort was identified on January 2, 2016, followed by 12 cases (detected by rRT-PCR and/or the serological algorithm) through the end of February 2016 (Fig 2). From the beginning of March to mid-June, which corresponds to the dry season and is typically a period of low transmission of *Aedes*-borne viruses in the study area [24], 20 cases were identified. Zika cases were detected in greater numbers starting in mid-June, and the majority of the cases in the epidemic (*n =* 516; 92.1%) occurred by the end of September. Twelve cases occurred between October 2016 and February 2017. The overall incidence of Zika in our study population was 14.0 cases per 100 person-years (95% CI: 12.9, 15.2), and the weekly incidence peaked in the last week of July, at 123.5 cases per 100 person-years (95% CI: 100.0, 152.6) (Table 2). During the same period, a total of 17 symptomatic DENV infections were identified by rRT-PCR and/or virus isolation, including 3 co-infections with ZIKV. All DENV infections were caused by the DENV-2 serotype.

**Table 2:** Incidence of ZIKV infection and Zika cases in participants aged 2–14 years, District II of Managua, January 2016–February 2017.

Analysis	Zika cases*			ZIKV infections^†^		
	Person-years	Cases	Incidence per 100 person-years (95% CI)	Person-years	Infections	Incidence per 100 person-years (95% CI)
**Overall**	3,996.6	560	14.0 (12.9, 15.2)	3,707.9	1,356	36.5 (34.7, 38.6)
**By sex**						
Female	1,985.6	309	15.6 (13.9, 17.4)	1,859.1	722	38.8 (36.1, 41.8)
Male	2,011.0	251	12.5 (11.0, 14.1)	1,848.8	634	34.3 (31.7, 37.1)
**By age (years)**						
2–5	1,236.7	128	10.4 (8.7, 12.3)	1,358.1	391	28.8 (26.1, 31.8)
6–9	1,316.5	205	15.6 (13.6, 17.9)	1,313.5	488	37.2 (34.0, 40.6)
10–14	1,443.5	227	15.7 (13.8, 17.9)	1,036.3	477	46.0 (42.1, 50.4)

*Symptomatic ZIKV infections detected from January 2016 to February 2017.

^†^Symptomatic and inapparent ZIKV infections detected from January 2016 to February 2017 in children who provided annual samples in 2015, 2016, and 2017, and from March 2016 to February 2017 in children who provided annual samples in 2016 and 2017 but not in 2015.

ZIKV, Zika virus.

### Incidence of Zika cases and total ZIKV infections

We then calculated the incidence of symptomatic ZIKV infections (Zika cases) according to the demographic characteristics of the participants. Zika incidence was higher in girls than in boys (crude IRR: 1.22; 95% CI: 1.02, 1.45; *p =* 0.028), with a rate of 15.6 cases per 100 person-years (95% CI: 13.9, 17.4) in girls and 12.5 cases per 100 person-years (95% CI: 11.0, 14.1) in boys (Tables 2 and 3). Zika incidence also increased with age group (Tables 2 and 3). Of the 3,893 children in the cohort, 3,296 children provided paired annual samples in 2016 and 2017. The incidence of total ZIKV infections (symptomatic and inapparent) in these children was 36.5 (95% CI: 34.7, 38.6) infections per 100 person-years. Girls had 1.15 (95% CI: 1.02, 1.31; *p =* 0.028) times the rate of ZIKV infection of boys. ZIKV infection incidence increased with increasing age (Tables 2 and 3).

**Table 3:** Effect of demographic characteristics and recent and prior DENV infection on risk of symptomatic ZIKV infection, ZIKV infection, and symptomatic presentation given ZIKV infection.

Analysis	Crude IRR	*p-*Value	IRR adjusted for age and sex	*p-*Value	Multivariable model IRR	*p-*Value
**Risk of symptomatic ZIKV infection**						
Female sex	1.22 (1.02, 1.45)	0.028	**1.21 (1.02, 1.44)**	0.031	**1.21 (1.01, 1.44)**	0.033
Age (years)	**1.05 (1.02, 1.07)**	<0.001	**1.04 (1.02, 1.07)**	<0.001	**1.07 (1.05, 1.10)**	<0.001
Recent DENV infection	**0.61 (0.38, 0.98)**	0.042	**0.57 (0.35, 0.92)**	0.020	0.80 (0.47, 1.34)	0.393
Prior DENV infection	**0.77 (0.62, 0.96)**	0.020	**0.60 (0.47, 0.76)**	<0.001	**0.63 (0.48, 0.81)**	<0.001
**Risk of ZIKV infection**						
Female sex	**1.15 (1.02, 1.31)**	0.028	**1.14 (1.00, 1.29)**	0.047	**1.14 (1.00, 1.29)**	0.045
Age (years)	**1.09 (1.07, 1.11)**	<0.001	**1.09 (1.07, 1.11)**	<0.001	**1.09 (1.07, 1.12)**	<0.001
Recent DENV infection	0.93 (0.69, 1.25)	0.613	0.83 (0.61, 1.12)	0.219	0.84 (0.61, 1.16)	0.295
Prior DENV infection	**1.28 (1.11, 1.48)**	<0.001	0.94 (0.81, 1.11)	0.482	0.98 (0.82, 1.16)	0.809
**Risk of symptomatic presentation given ZIKV infection**						
Female sex	1.16 (0.92, 1.45)	0.211	1.15 (0.92, 1.45)	0.215	1.14 (0.91, 1.44)	0.244
Age (years)	1.01 (0.97, 1.04)	0.748	1.00 (0.97, 1.04)	0.779	1.03 (0.99, 1.07)	0.092
Recent DENV infection	0.62 (0.32, 1.21)	0.162	0.62 (0.32, 1.20)	0.157	0.87 (0.43, 1.77)	0.698
Prior DENV infection	**0.68 (0.51, 0.90)**	0.008	**0.60 (0.44, 0.82)**	0.001	**0.62 (0.44, 0.86)**	0.004

IRR values in bold are statistically significant.

DENV, dengue virus; IRR, incidence rate ratio; ZIKV, Zika virus.

### Effect of prior DENV infection on Zika and ZIKV infection incidence

A total of 3,027 children in the cohort had known DENV infection histories and were included in the models examining the effects of prior DENV infection on symptomatic Zika rates. Of these children, 743 (24.5%) had experienced at least 1 prior DENV infection in the cohort, 176 (5.8%) had experienced a recent DENV infection, and 2,284 children (75.5%) were DENV-naïve (S1 Table). Rates of documented DENV infection increased with age and were similar among males and females. Children with prior DENV infection had a significantly lower incidence of symptomatic Zika and a lower rate of symptomatic presentation given ZIKV infection than DENV-naïve children. In multivariable models adjusting for age and sex, both having a prior DENV infection and having a recent DENV infection were significantly negatively associated with the incidence of symptomatic Zika. However, when both prior DENV infection and recent DENV infection were included in the model, only prior DENV infection remained significant (IRR: 0.63; 95% CI: 0.48, 0.81; *p <* 0.005).

Of the 3,027 children with known DENV infection histories, 2,659 children contributed paired annual serum samples for 2016 and 2017 and were thus included in the ZIKV infection analysis. Of these children, 574 (21.6%) had 1 or more prior DENV infections, 134 (5%) had experienced a recent DENV infection, and 2,085 (78.4%) were DENV-naïve (S2 Table). In multivariable Poisson models, the rate of ZIKV infection was not significantly associated with prior DENV infection status when adjusted for age, sex, and recent DENV infection (IRR: 0.98; 95% CI: 0.82, 1.16; *p =* 0.809). Further, among ZIKV infections, children with prior DENV infection had 0.62 (95% CI: 0.44, 0.86; *p =* 0.004) times the rate of symptomatic Zika presentation compared to DENV-naïve children when adjusted for recent DENV infection, age, and sex (Table 3). Among ZIKV infections, primary DENV immunity was associated with a decreased rate of symptomatic ZIKV infection compared to being DENV-naïve (IRR: 0.56, 95% CI: 0.38, 0.83; *p =* 0.004). While secondary DENV immunity was not significantly associated with symptomatic Zika (IRR: 0.66, 95% CI: 0.44, 1.01; *p =* 0.056) compared to being DENV-naïve, the point estimate was similar to that of primary DENV immunity.

### Early epidemic growth scaling and reproduction numbers

Using the early growth phase comprising the first few weeks of the major Zika epidemic, which began in June 2016, we estimated the scaling of growth parameter (*p*) at 0.8 (95% CI: 0.2, 1.0) based on the first 5 weeks of the epidemic peak and 0.9 (95% CI: 0.7, 1.0) based on the first 8 weeks of epidemic growth (Table 4). Thus, the growth scaling uncertainty for this epidemic, affecting a largely susceptible population, includes exponential growth dynamics (i.e., *p =* 1). Based on the GGM, we estimated *R*_e_ at 3.3 (95% CI: 1.3, 5.3) and 3.4 (95% CI: 2.4, 4.7) based on 5 and 8 weeks of data of the early growth phase, respectively, and assuming a generation interval of 20 days (SD = 7.4 days) [40,41].

**Table 4:** Mean estimates and corresponding 95% confidence intervals for the effective reproduction number during the early growth phase of the Zika epidemic.

Parameter	5-Week ascending phase	8-Week ascending phase
Reproduction number, *R*_e_*	3.3 (1.3, 5.3)	3.4 (2.4, 4.7)
Growth rate, *r*	0.2 (0.1, 0.4)	0.1 (0.1, 0.2)
Scaling of growth parameter, *p*	0.8 (0.2, 1.0)	0.9 (0.7, 1.0)

*We assumed a generation interval of 20 days and SD of 7.4 days [41].

### Discussion

In this study, we took advantage of our long-standing dengue cohort study to characterize ZIKV dynamics during the Zika epidemic in Managua, Nicaragua, from its introduction in January 2016 to February 2017. We found a high incidence of both symptomatic and total ZIKV infections and estimated the effective reproduction number during the early growth phase of the epidemic to be 3.3–3.4. Girls and older children had a higher risk of ZIKV infection. Notably, PDCS children with prior DENV infection had a lower risk of developing symptoms when infected by ZIKV.

Using a simple growth model that incorporates the possibility of sub-exponential growth dynamics via the parameter *p*, we were able to derive estimates of *R*_e_ from the early epidemic growth phase. Our estimates of *R*_e_ for the Zika epidemic in Managua lie within the range of estimates for Zika outbreaks in Colombia (2–5) [39,42,43] and in the South Pacific (1.5–5.8) [44–46] and are slightly higher than estimates from Brazil (1.8–3.0) [47,48].

Flaviviruses are known to induce both virus type-specific and cross-reactive immune responses [49]. Early evidence of the cross-reactivity of ZIKV- and DENV-induced immune responses was observed during the 2007 Yap Island Zika outbreak, in particular in patients thought to have been previously DENV-exposed [6]. More recently, studies have shown that monoclonal antibodies from DENV-exposed persons are able to neutralize ZIKV in vitro and in mouse models [16,18,50]. In rhesus macaques, ZIKV pathogenesis was unaffected by prior DENV exposure (although ZIKV viremia was shorter in pre-exposed animals [20]), and in humans with acute, symptomatic ZIKV infection, no major changes in viral load or cytokine levels were reported in DENV-pre-exposed versus DENV-naïve patients [21]. Here, we show that children with a prior DENV infection have a lower risk of being symptomatic when infected by ZIKV after adjusting for sex and age, suggesting that previous DENV immunity may be protective against Zika. However, studies conducted using sera from humans exposed to DENV suggest that cross-reactive neutralizing responses induced by DENV against ZIKV are neither as strong nor as durable as those induced against heterologous DENV types [50–52]. Thus, we analyzed the risk of symptomatic presentation given ZIKV infection in children with a recent (2015/2016 season) DENV infection. The IRR estimate was 0.57 (95% CI: 0.35, 0.92; *p =* 0.020) when adjusted for age and sex; however, including prior DENV infection in the model increased the IRR estimate to 0.80 (95% CI: 0.47, 1.34; *p =* 0.393). It is important to note that the incidence of DENV infection in the PDCS during the 2015/2016 season was low, which limits the power of the analysis. Additionally, cross-protection from a prior DENV infection might be provided through other antibody-mediated responses (e.g., antibody-dependent cellular cytotoxity) and/or through cross-protective CD8^+^ T cell responses [19,53,54].

The strengths of our study include its large size and prospective nature. In addition, given that our cohort study has been ongoing since 2004, with both symptomatic and inapparent DENV infections being characterized, we are uniquely positioned to examine the effect of prior DENV infection on Zika risk. Our study has several limitations; first, it is restricted to children and thus we cannot comment on the effect of prior DENV infection in adults. Second, we are constrained by the epidemiology of our site, including the limited number of DENV infections in the years immediately preceding ZIKV introduction. Third, due to incomplete DENV infection histories and/or the lack of paired annual serum samples, a subset of the cohort was excluded from the DENV immunity analyses. Fourth, for the identification of symptomatic ZIKV infections, we relied on participants to present at the health center; thus, it is possible that we misclassified some infections as inapparent that were truly symptomatic. If presentation at the health center among children with symptomatic infections was related to DENV immunity, this could have biased our analyses. Fifth, determination of rRT-PCR-negative symptomatic ZIKV infections and inapparent ZIKV infections relied on an algorithm that combined several ZIKV and DENV serological test results and on the ZIKV NS1 BOB ELISA, respectively. Although the algorithm was developed to maximize its diagnostic value and the ZIKV NS1 BOB assay has been characterized in different populations [34,35,55], we cannot exclude that several symptomatic and inapparent infections were misclassified. Sixth, risk of exposure to DENV and ZIKV are not independent, as the 2 viruses are both transmitted by the same mosquito and the underlying risk of exposure to arboviruses likely varies by home and school location as well as child. Underlying arboviral risk is a potential unmeasured confounder in our study. This may have biased our infection analysis towards the null, as children who have previously been exposed to DENV may be generally at higher risk for arboviral infections than children who have not been exposed to DENV. In part to address this underlying relationship between DENV exposure risk and ZIKV exposure risk, we performed the analysis examining symptomatic presentation among those with ZIKV infections, which removes this potential source of bias. Importantly, the effect estimate for prior DENV immunity obtained in this analysis was very similar to that obtained in the symptomatic Zika analysis, indicating that this potential source of bias did not significantly impact the symptomatic Zika analysis.

In summary, we documented a high incidence of ZIKV infection and Zika disease in children in Managua, Nicaragua, during the 2016 epidemic. We found that prior DENV infection was associated with a decreased risk of Zika symptoms in individuals exposed to ZIKV. This supports that DENV immunity may be protective for symptomatic ZIKV infection. Our findings might be generalizable to other locations that have had DENV circulation in the years preceding ZIKV circulation, including a great majority of countries in the Americas. Conversely, it is possible that this cross-reactivity in the human immune response among closely related flaviviruses may be responsible for the dramatic decrease in dengue cases during and right after the Zika epidemic throughout the Americas. More research is needed to address the possible immunological mechanism(s) of cross-protection between ZIKV and DENV and whether DENV immunity may modulate other ZIKV infection outcomes such as neurological or congenital syndromes.

## Supporting information

S1 Appendix Protocol of the Pediatric Dengue Cohort Study (PDCS). (PDF)

S2 Appendix STROBE checklist for cohort studies. (DOCX)

S1 Table Characteristics of the participants included in the analysis of the effect of prior DENV infection on the risk of symptomatic ZIKV infection. (DOCX)

S2 Table Characteristics of the participants included in the analysis of the effect of prior DENV infection on the risk of ZIKV infection and the risk of symptomatic presentation among those with ZIKV infection. (DOCX)

## Abbreviations

BOB: blockade-of-binding
DENV: dengue virus
GBS: Guillain–Barré syndrome
GGM: generalized-growth method
HCSFV: Health Center Sócrates Flores Vivas
iELISA: inhibition ELISA
IRR: incidence rate ratio
MAC-ELISA: IgM antibody capture ELISA
OFI: other febrile illness
PDCS: Pediatric Dengue Cohort Study
rRT-PCR: real-time reverse transcription PCR
ZIKV: Zika virus
