# Supplementary material for: Prior dengue virus infection and risk of Zika: A pediatric cohort in Nicaragua
Source: PLoS Med. 2019 Jan 22;16(1):e1002726. doi: 10.1371/journal.pmed.1002726 (PMC6342296; doi:10.1371/journal.pmed.1002726)
Supplement: S1 Appendix — (PDF) [file pmed.1002726.s001.pdf]

PROTOCOL
Biomedical Non-Exempt
Berkeley

Protocol # 2010-09-2245
Date Printed: 10/05/2018

Protocol Title: Pediatric Cohort Study of Dengue Transmission in Nicaragua

Protocol Type: Biomedical Non-Exempt

Date Submitted: Draft

Approval Period: Draft

Important Note: This Print View may not reflect all comments and contingencies for approval. Please check the comments section of the online protocol.
Questions that appear to not have been answered may not have been required for this submission. Please see the system application for more details.

---

* * * Personnel Information * * *

Enter all UC Berkeley study personnel (if not previously entered) and relevant training information. Please read Personnel Titles and Responsibilities: Roles in eProtocol before completing this section.

Note: The Principal Investigator or Faculty Sponsor, Co-Principal Investigator, Student or Postdoctoral Investigator, Administrative Contact, and Other Contact can EDIT and SUBMIT. Other Personnel can only VIEW the protocol.

Principal Investigator or Faculty Sponsor

Name of Principal Investigator	Degree (e.g., MS/PhD)	Title
Eva HARRIS	PhD	Professor
**Email**	**Phone**	**Fax**
eharris@berkeley.edu	+1 510 642-4845	+1 510 642-6350
**Department Name**	**Mailing Address**	
Pub Hlth-Infectious Diseases	94720-7354	

UCB status (select all that apply):

X	Faculty		Postdoc		Grad		Undergrad		Other

Faculty (with some exceptions), staff, and students engaged in human subjects research must complete either the biomedical or social-behavioral human research course through the online Collaborative Institutional Training Initiative (CITI), depending upon which is most germane to the research. ALL PIs on an NIH award are required to complete either CITI or NIH Training. See Training and Education for more information.

If applicable, please insert date (mm/dd/yy) of completion in appropriate box(es) below:

CITI	NIH	Other Training (title & date completed)
09/02/11	10/24/10	

Student or Postdoctoral Investigator

Note: All Student/Postdoc Investigators must have a Faculty Sponsor who will serve as the "responsible researcher." If NOT a student or postdoc project, enter the student(s) and/postdoc(s) under Other Personnel below.

Name of Student/Postdoc Investigator	Degree	Title
Raquel Burger-Calderon	PhD MPH	
**Email**	**Phone**	**Fax**

Protocol Title: Pediatric Cohort Study of Dengue Transmission in Nicaragua
Protocol Type: Biomedical Non-Exempt
Date Submitted: Draft
Approval Period: Draft
Important Note: **This Print View may not reflect all comments and contingencies for approval. Please check the comments section of the online protocol.**
Questions that appear to not have been answered may not have been required for this submission. Please see the system application for more details.

---

rqburger@gmail.com 9195905792

Department Name
Pub Hlth-Infectious Diseases

Mailing Address
19931 N River Rd
Alva, FL 33920

UCB status (select all that apply):

	**Faculty**	X	**Postdoc**		**Grad**		**Undergrad**		**Other**	

Faculty (with some exceptions), staff, and students engaged in human subjects research must complete either the biomedical or social-behavioral human research course through the online Collaborative Institutional Training Initiative (CITI), depending upon which is most germane to the research. ALL PIs on an NIH award are required to complete either CITI or NIH Training. See Training and Education for more information.

If applicable, please insert date (mm/dd/yy) of completion in appropriate box(es) below:

**CITI**	**NIH**	**Other Training (title & date completed)**
05/31/16		

Administrative Contact

**Name of Administrative Contact**	**Degree**	**Title**
Josefina COLOMA		PROJECT SCIENTIST
**Email**	**Phone**	**Fax**
colomaj@berkeley.edu	+1 510 643-9773	+1 510 642-6350
**Department Name**	**Mailing Address**	
Pub Hlth-Infectious Diseases	94720-7360	

UCB status (select all that apply):

	**Faculty**		**Postdoc**		**Grad**		**Undergrad**		**Other**	

Other Contact

**Name of Other Contact**	**Degree**	**Title**
Anna Gajewski	MPH	
**Email**	**Phone**	**Fax**

Protocol Title: Pediatric Cohort Study of Dengue Transmission in Nicaragua

Protocol Type: Biomedical Non-Exempt

Date Submitted: **Draft**

Approval Period: **Draft**

Important Note: **This Print View may not reflect all comments and contingencies for approval. Please check the comments section of the online protocol.**
Questions that appear to not have been answered may not have been required for this submission. Please see the system application for more details.

---

agajewski@icsnicaragua.org

**Department Name**	**Mailing Address**
SPH Administration	

UCB status (select all that apply):

	**Faculty**		**Postdoc**		**Grad**		**Undergrad**	X	**Other**		**Study Coordinator**

Protocol Title: Pediatric Cohort Study of Dengue Transmission in Nicaragua
Protocol Type: Biomedical Non-Exempt
Date Submitted: Draft
Approval Period: Draft
Important Note: This Print View may not reflect all comments and contingencies for approval. Please check the comments section of the online protocol.
Questions that appear to not have been answered may not have been required for this submission. Please see the system application for more details.

---

* * * Vulnerable Subject Checklist * * *

Vulnerable Subject Checklist

Yes	No	
Y		Children/Minors
	N	Prisoners
	N	Pregnant Women
	N	Fetuses
	N	Neonates
Y		Educationally Disadvantaged
Y		Economically Disadvantaged
	N	Cognitively Impaired
	N	Other (i.e., any vulnerable subject population(s) not specified above)

**Protocol Title:**	Pediatric Cohort Study of Dengue Transmission in Nicaragua
**Protocol Type:**	Biomedical Non-Exempt
**Date Submitted:**	Draft
**Approval Period:**	Draft
**Important Note:**	This Print View may not reflect all comments and contingencies for approval. Please check the comments section of the online protocol.<br>Questions that appear to not have been answered may not have been required for this submission. Please see the system application for more details.

---

* * * Study Sites * * *

Study Sites

Select all study sites where data collection via subject interaction will take place:

International

X International Site(s) (specify country, region, and township or village)

Managua, Nicaragua

Local

X UC Berkeley

UC Davis

UC Irvine

UC Los Angeles

UC Merced

UC Riverside

UC San Diego

UC San Francisco

UC Santa Barbara

UC Santa Cruz

Lawrence Berkeley National Laboratory

Alameda Unified School District (specify schools below)

Berkeley Unified School District (specify schools below)

Oakland Unified School District (specify schools below)

Other (Specify other Study Sites)

Protocol Title: Pediatric Cohort Study of Dengue Transmission in Nicaragua
Protocol Type: Biomedical Non-Exempt
Date Submitted: Draft
Approval Period: Draft
Important Note: **This Print View may not reflect all comments and contingencies for approval. Please check the comments section of the online protocol.**
Questions that appear to not have been answered may not have been required for this submission. Please see the system application for more details.

---

* * * General Checklist * * *

General Checklist

Yes	No	
Y		Is the research receiving any federal funding (e.g., NIH, NSF, DOD, etc.)?
	N	Is another campus relying on UC Berkeley for IRB review by means of the UC System Memorandum of Understanding (MOU)?
	N	Is another institution relying on UC Berkeley for IRB review by means of an Inter-institutional IRB Authorization Agreement?
	N	Will subjects be compensated for participation?
Y		Is this protocol administratively supported by Campus Shared Services Team 9?
	N	Does this research fall under FDA regulations?
Y		Any use of human blood, body fluids, tissues, or cells (including cell lines)* by drawing samples, accepting samples already drawn, receiving samples from any source, or in any other way?
		If yes, Lab Location: Managua, Nicaragua; SPH, UCB
		And Biological Use Authorization (BUA) #(s): 52 (UCB)
Y		Will biological specimens be stored for future research projects?
Y		Will specimens be sent out of UCB as part of a research agreement?
	N	Will proprietary drug or device testing be done?
	N	Any use of embryonic stem cells? *NOTE: If research involves embryonic stem cells, see UCB Stem Cell Policy and Committee.
	N	Any use of medical devices or equipment cleared/approved for marketing?
	N	Any use of any experimental or investigational devices or equipment (i.e., not cleared/approved for marketing?)
	N	Any use of commercially available drugs, reagents, or other chemicals administered to subjects (even if drugs themselves are not being studied)?
	N	Any use of investigational drugs, reagents, or chemicals (i.e., not cleared/approved for marketing)?

Protocol Title: Pediatric Cohort Study of Dengue Transmission in Nicaragua
Protocol Type: Biomedical Non-Exempt
Date Submitted: Draft
Approval Period: Draft
Important Note: This Print View may not reflect all comments and contingencies for approval. Please check the comments section of the online protocol.
Questions that appear to not have been answered may not have been required for this submission. Please see the system application for more details.

---

* * * Funding * * *

Funding Checklist

If the research is not funded, check the "Not Funded" box below. If the research is funded, add the funding source to the appropriate table below.

NOTE: Only the Principal Investigator (PI) of the grant or subcontract can add his or her own SPO Funding information in this section. The PI of the grant must also be listed in the Personnel Information section of the protocol in one of the following roles: Principal Investigator or Faculty Sponsor, Student or Postdoctoral Investigator, Co-Principal Investigator, Administrative Contact, or Other Contact. Training Grants can be added by anyone in one of the aforementioned roles. For step-by-step instructions, see Add SPO Funding Quick Guide

Not Funded

SPO - Funding

SPO ID	Sponsor	Sponsor Award ID	Prime Sponsor

SPO - Funding

SPO ID	20100249
Sponsor Award ID	
Sponsor	McMasters University (incl Hamilton Health Sciences Corp)
Prime Sponsor	NIH National Institute of Allergy & Infectious Diseases
Funding Status	Funded
Principal Investigator	Harris, Eva
Co-Investigator (s)	
Admin Unit	School of Public Health
Project Title	Dengue Population Genetics Program
Amount	460500
Start	2012-06-01 00:00:00.0
End	2015-05-31 00:00:00.0
Subcontracts	No

**Protocol Title:**	Pediatric Cohort Study of Dengue Transmission in Nicaragua
**Protocol Type:**	Biomedical Non-Exempt
**Date Submitted:**	**Draft**
**Approval Period:**	**Draft**
**Important Note:**	**This Print View may not reflect all comments and contingencies for approval. Please check the comments section of the online protocol. Questions that appear to not have been answered may not have been required for this submission. Please see the system application for more details.**

**SPO ID**	20120052
**Sponsor Award ID**	
**Sponsor**	Colorado State University
**Prime Sponsor**	NIH National Institutes of Health - Miscellaneous
**Funding Status**	Pending
**Principal Investigator**	Harris, Eva
**Co-Investigator (s)**	
**Admin Unit**	School of Public Health
**Project Title**	Metabolomic Small Molecule Bimarkers for Noninvasive Dengue Diagnosis and Prognosis
**Amount**	$918,034
**Start**	5/1/2012
**End**	4/30/2017
**Subcontracts**	Yes

**SPO ID**	20113432
**Sponsor Award ID**	
**Sponsor**	Sustainable Sciences Institute
**Prime Sponsor**	NIH National Institutes of Health - Miscellaneous
**Funding Status**	Funded
**Principal Investigator**	Harris, Eva
**Co-Investigator (s)**	
**Admin Unit**	Dean's Office, Public Health
**Project Title**	Characterization of 3rd & 4th Denque Virus Infections in a Pediatric Cohort Study
**Amount**	$109,644
**Start**	5/1/2012
**End**	4/30/2017
**Subcontracts**	No

**SPO ID**	20132635

**Protocol Title:**	Pediatric Cohort Study of Dengue Transmission in Nicaragua
**Protocol Type:**	Biomedical Non-Exempt
**Date Submitted:**	**Draft**
**Approval Period:**	**Draft**
**Important Note:**	**This Print View may not reflect all comments and contingencies for approval. Please check the comments section of the online protocol. Questions that appear to not have been answered may not have been required for this submission. Please see the system application for more details.**

**Sponsor Award ID**	
**Sponsor**	UC San Francisco
**Prime Sponsor**	Bill & Melinda Gates Foundation
**Funding Status**	Pending
**Principal Investigator**	Harris, Eva
**Co-Investigator (s)**	Coloma, Josefina
**Admin Unit**	School of Public Health
**Project Title**	Fighting Infections through Research, Science and Technology (FIRST)
**Amount**	$557,346
**Start**	4/1/2013
**End**	3/31/2015
**Subcontracts**	Yes

**SPO ID**	20150447
**Sponsor Award ID**	
**Sponsor**	Colorado State University
**Prime Sponsor**	NIH National Institute of Allergy & Infectious Diseases
**Funding Status**	Funded
**Principal Investigator**	Eva Harris
**Co-Investigator (s)**	
**Admin Unit**	SPH Divisional Rsrch and Cntrs
**Project Title**	Metaboloic Small Molecule Bimarkers for Noninvasive Dengue Diagnosis and Prognosis
**Amount**	$242,694
**Start**	6/1/2014
**End**	5/31/2015
**Subcontracts**	No

**SPO ID**	036285-002
**Sponsor Award ID**	8032sc

**Protocol Title:**	Pediatric Cohort Study of Dengue Transmission in Nicaragua
**Protocol Type:**	Biomedical Non-Exempt
**Date Submitted:**	**Draft**
**Approval Period:**	**Draft**
**Important Note:**	**This Print View may not reflect all comments and contingencies for approval. Please check the comments section of the online protocol.<br>Questions that appear to not have been answered may not have been required for this submission. Please see the system application for more details.**

**Sponsor**	UC San Francisco
**Prime Sponsor**	Carlos Slim Health Institute
**Funding Status**	Active
**Principal Investigator**	Harris, Eva
**Co-Investigator (s)**	Coloma, Josefina
**Admin Unit**	School of Public Health
**Project Title**	Fighting Infections through Research, Science and Technology (FIRST)
**Amount**	$132,207
**Start**	12/12/2013
**End**	3/31/2015
**Subcontracts**	No

**SPO ID**	20143099
**Sponsor Award ID**	
**Sponsor**	Sustainable Sciences Institute
**Prime Sponsor**	NIH National Institutes of Health - Miscellaneous
**Funding Status**	Pending
**Principal Investigator**	Harris, Eva
**Co-Investigator (s)**	
**Admin Unit**	SPH Divisional Rsrch and Cntrs
**Project Title**	Supplement for Characterization of 3rd & 4th Dengue virus Infections in a Pediatric Cohort Study
**Amount**	$19,602
**Start**	7/1/2014
**End**	6/30/2017
**Subcontracts**	No

**SPO ID**	20150842
**Sponsor Award ID**	
**Sponsor**	Icahn School of Medicine at Mount Sinai
**Prime Sponsor**	NIH National Institutes of Health - Miscellaneous

**Protocol Title:**	Pediatric Cohort Study of Dengue Transmission in Nicaragua
**Protocol Type:**	Biomedical Non-Exempt
**Date Submitted:**	**Draft**
**Approval Period:**	**Draft**
**Important Note:**	**This Print View may not reflect all comments and contingencies for approval. Please check the comments section of the online protocol. Questions that appear to not have been answered may not have been required for this submission. Please see the system application for more details.**

**Funding Status**	Pending
**Principal Investigator**	Harris, Eva
**Co-Investigator (s)**	
**Admin Unit**	SPH Divisional Rsrch and Cntrs
**Project Title**	Dengue Human Immunology Project Consortium (DHIPC)
**Amount**	$1,360,613
**Start**	7/1/2015
**End**	6/30/2020
**Subcontracts**	No

**SPO ID**	20150240
**Sponsor Award ID**	
**Sponsor**	NIH National Institute of Allergy & Infectious Diseases
**Prime Sponsor**	
**Funding Status**	Pending
**Principal Investigator**	Harris, Eva
**Co-Investigator (s)**	
**Admin Unit**	SPH Divisional Rsrch and Cntrs
**Project Title**	Protective Immunity Following Dengue Virus Natural Infections and Vaccination
**Amount**	$12,790,582
**Start**	7/1/2015
**End**	6/30/2020
**Subcontracts**	Yes

**SPO ID**	034192-002
**Sponsor Award ID**	IR1200-1
**Sponsor**	Sustainable Sciences Institute
**Prime Sponsor**	NIH National Institute of Allergy & Infectious Diseases
**Funding Status**	Active

**Protocol Title:**	Pediatric Cohort Study of Dengue Transmission in Nicaragua
**Protocol Type:**	Biomedical Non-Exempt
**Date Submitted:**	Draft
**Approval Period:**	Draft
**Important Note:**	**This Print View may not reflect all comments and contingencies for approval. Please check the comments section of the online protocol. Questions that appear to not have been answered may not have been required for this submission. Please see the system application for more details.**

**Principal Investigator**	Eva Harris
**Co-Investigator (s)**	
**Admin Unit**	SPH Divisional Rsrch and Cntrs
**Project Title**	Characterization of 3rd & 4th Denque Virus Infections in a Pediatric Cohort Study
**Amount**	$141,918
**Start**	6/28/2012
**End**	5/31/2018
**Subcontracts**	No

**SPO ID**	20183388
**Sponsor Award ID**	
**Sponsor**	Icahn School of Medicine at Mount Sinai
**Prime Sponsor**	NIH National Institutes of Health - Miscellaneous
**Funding Status**	Pending
**Principal Investigator**	Eva Harris
**Co-Investigator (s)**	
**Admin Unit**	SPH Divisional Rsrch and Cntrs
**Project Title**	Immune Variation from Infancy to Adolescence: Dissecting the Effects of Age, Gender, and Individuality
**Amount**	$313,986
**Start**	6/1/2018
**End**	5/31/2019
**Subcontracts**	No

Funding - Other

Protocol Title: Pediatric Cohort Study of Dengue Transmission in Nicaragua

Protocol Type: Biomedical Non-Exempt

Date Submitted: Draft

Approval Period: Draft

Important Note: This Print View may not reflect all comments and contingencies for approval. Please check the comments section of the online protocol.
Questions that appear to not have been answered may not have been required for this submission. Please see the system application for more details.

**Protocol Title:**	Pediatric Cohort Study of Dengue Transmission in Nicaragua
**Protocol Type:**	Biomedical Non-Exempt
**Date Submitted:**	**Draft**
**Approval Period:**	**Draft**
**Important Note:**	**This Print View may not reflect all comments and contingencies for approval. Please check the comments section of the online protocol.**<br>**Questions that appear to not have been answered may not have been required for this submission. Please see the system application for more details.**

---

* * * Expedited Paragraphs * * *

Request for Expedited Review

An expedited review procedure consists of a review of research involving human subjects by the IRB Chair, or by one or more experienced reviewers designated by the Chairperson from among the members of the committees.

In order to be eligible for expedited review, ALL aspects of the research must include activities that (1) present no more than minimal risk to human subjects, and (2) involve only procedures included in one or more of the specific categories listed below.

If requesting Expedited Review, select one or more of the applicable paragraph(s) below.
(DO NOT select any paragraph(s) if your protocol does not qualify for expedited review. Protocols that do not qualify for expedited review will be reviewed by the full (convened) Committee.)

1. **Clinical studies of drugs and medical devices only when conditions (a) or (b) are met.**

   a) Research on drugs for which an investigational new drug application (21 CFR Part 312) is not required. (Note: Research on marketed drugs that significantly increases the risks or decreases the acceptability of the risks associated with the use of the product is not eligible for expedited review.)

   b) Research on medical devices for which

   i) an investigational device exemption application (21 CFR Part 812) is not required; or
   ii) the medical device is cleared/approved for marketing and the medical device is being used in accordance with its cleared/approved labeling.

X 2. **Collection of blood samples by finger stick, heel stick, ear stick, or venipuncture as follows:**

   a) From healthy, non-pregnant adults who weigh at least 110 pounds. For these subjects, the amounts drawn may not exceed 550 ml in an 8 week period and collection may not occur more frequently than 2 times per week; or

   b) From other adults and children, considering the age, weight, and health of the subjects, the collection procedure, the amount of blood to be collected, and the frequency with which it will be collected. For these subjects, the amount drawn may not exceed the lesser of 50 ml or 3 ml per kg in an 8 week period and collection may not occur more frequently than 2 times per week.

X 3. **Prospective collection of biological specimen for research purposes by non-invasive means.**

Examples:

a) hair and nail clippings in a non-disfiguring manner;
b) deciduous teeth at time of exfoliation or if routine patient care indicates a need for extraction;
c) permanent teeth if routine patient care indicates a need for extraction;
d) excreta and external secretions (including sweat);
e) uncannulated saliva collected either in an unstimulated fashion or stimulated by chewing gumbase or wax or by applying a dilute citric solution to the tongue;
f) placenta removed at delivery;
g) amniotic fluid obtained at the time of rupture of the membrane prior to or during labor;
h) supra- and subgingival dental plaque and calculus, provided the collection procedure is not more invasive than routine prophylactic scaling of the teeth and the process is accomplished in accordance with accepted prophylactic techniques;
i) mucosal and skin cells collected by buccal scraping or swab, skin swab, or mouth washings;
j) sputum collected after saline mist nebulization.

4. **Collection of data through non-invasive procedures (not involving general anesthesia or sedation) routinely employed in clinical practice, excluding procedures involving x rays or microwaves. Where medical devices are employed, they must be cleared/approved for marketing. (Studies intended to evaluate the safety and effectiveness of the medical device are not generally eligible for expedited review, including studies of cleared medical devices for new indications.)**

Examples:

a) physical sensors that are applied either to the surface of the body or at a distance and do not involve input of significant amounts of energy into the subject or an invasion of the subject's privacy;
b) weighing or testing sensory acuity;
c) magnetic resonance imaging;
d) electrocardiography, electroencephalography, thermography, detection of naturally occurring radioactivity, electroretinography, ultrasound, diagnostic infrared imaging, doppler blood flow, and echocardiography;
e) moderate exercise, muscular strength testing, body composition assessment, and flexibility testing where appropriate given the age, weight, and health of the individual.

5. **Research involving materials (data, documents, records, or specimens) that have been collected or will be collected solely for non-research purposes (such as medical treatment or diagnosis). (NOTE: Some research in this paragraph may be exempt from the HHS regulations for the protection of human subjects. 45 CFR 46.101(b)(4). This listing refers only to research that is not exempt.)**

---

6. **Collection of data from voice, video, digital, or image recordings made for research purposes.**

X 7. **Research on individual or group characteristics or behavior (including, but not limited to, research on perception, cognition, motivation, identity, language, communication, cultural beliefs or practices, and social behavior) or research employing survey, interview, oral history, focus group, program evaluation, human factors evaluation, or quality assurance methodologies. (NOTE: Some research in this category may be exempt from the HHS regulations for the protection of human subjects. 45 CFR 46.101(b)(2) and (b)(3). This listing refers only to research that is not exempt.)**

8. **Continuing review of research previously approved by the convened IRB as follows:**

   a) Where (i) the research is permanently closed to the enrollment of new subjects; (ii) all subjects have completed all research-related interventions; and (iii) the research remains active only for long-term follow-up of subjects; or

   b) Where no subjects have been enrolled and no additional risks have been identified; or

   c) Where the remaining research activities are limited to data analysis.

9. **Continuing review of research, not conducted under an investigational new drug application or investigational device exemption where categories two (2) through eight (8) do not apply but the IRB has determined and documented at a convened meeting that the research involves no greater than minimal risk and no additional risks have been identified.**

Protocol Title: Pediatric Cohort Study of Dengue Transmission in Nicaragua
Protocol Type: Biomedical Non-Exempt
Date Submitted: Draft
Approval Period: Draft
Important Note: **This Print View may not reflect all comments and contingencies for approval. Please check the comments section of the online protocol.**
Questions that appear to not have been answered may not have been required for this submission. Please see the system application for more details.

---

* * * Purpose, Background, Collaborative Research * * *

Old CPHS # (for Protocols approved before eProtocol) **2004-6-178**

Study Title

Pediatric Cohort Study of Dengue Transmission in Nicaragua

Complete each section. When a question is not applicable, enter "N/A". Do not leave any sections blank.

1. Purpose

Provide a brief explanation of the proposed research, including specific study hypothesis, objectives, and rationale.

The overall aim of the project is to maintain a pediatric cohort in Nicaragua to characterize the natural history of dengue transmission and the range of clinical manifestations over time and to establish a potential trial site for antiviral therapies or a safe tetravalent dengue vaccine.

The specific aims of this project are to:
A. Prospectively determine the age- and serotype-specific incidence of symptomatic and asymptomatic dengue virus infection in children at highest risk of primary or secondary infection.
B. Characterize symptoms and disease spectrum and provide appropriate medical attention to all suspected symptomatic dengue cases within the study population.
C. Isolate representative samples of circulating dengue viruses and maintain a biobank of viruses.
D. Maintain a biobank of clinical specimens (serum/plasma, white blood cells, host DNA, urine and saliva) from symptomatic and asymptomatic infected persons linked to clinical information for use by collaborators.
E. Guarantee good clinical and laboratory practices through rigorous quality control.

2. Background

Give relevant background (e.g., summarize previous/current related studies) on condition, procedure, product, etc. under investigation, including citations if applicable (attach bibliography in Attachments section).

Dengue is the most important mosquito-borne viral disease affecting humans, caused by the four serotypes of dengue virus (DENV). Dengue fever (DF) and dengue hemorrhagic fever/dengue shock syndrome (DHF/DSS) have emerged as major public health problems, particularly in Southeast Asia and Latin America. According to the World Health Organization, an estimated 50 million cases of DF and 250,000-500,000 cases of DHF/DSS occur annually, with 3 billion people in over 100 countries at risk for infection. To date, no antiviral therapy or vaccine exists and case management relies on supportive treatment only. Dengue control relies on management of the Aedes aegypti and Ae. albopictus mosquito populations. An efficient, tetravalent vaccine would dramatically improve the fate of thousands of children and adults whose lives are affected by this disease, as would antivirals that could reduce the occurrence or

severity of symptomatic dengue. Cohorts need to be established in different areas of the world, and the appropriate field site infrastructure must be created in preparation for trials of candidate tetravalent vaccines and drugs currently in the pipeline. In addition, such studies allow numerous critical questions to be addressed regarding the protective and potentially detrimental elements of the immune response to DENV, as well as issues concerning DENV fitness and evolution.

Since the incidence of dengue has increased dramatically in the Americas in the last two decades, and Managua, Nicaragua, has experienced annual epidemics of considerable magnitude but relatively manageable proportions, we chose this city as an ideal site for a cohort study. In 2004, a Pediatric Dengue Vaccine Initiative-sponsored (PDVI) cohort was successfully established in District II of Managua, Nicaragua, and has been several times since then.

Chikungunya virus (CHIKV) is a re-emerging, mosquito-transmitted arthritogenic alphavirus that causes both endemic and explosive epidemics of debilitating rheumatic disease. In October 2013, the first autochthonous cases of chikungunya fever occurred on the Caribbean Island of St. Martin. In 2014, the virus has spread throughout the Caribbean, Central America, including Nicaragua, and to a lesser extent to South America and the United States. CHIKV-induced disease is commonly characterized by fever, and intense pain and inflammation in muscles, joints, and tendons. In May 2015, the Pan American Health Organization/World Health Organization published an alert describing the first outbreak of Zika fever in continental South America (specifically in Northeastern Brazil). Zika fever is caused by Zika virus (ZIKV), is similar in clinical presentation to dengue and chikungunya, and is transmitted by the same Aedes mosquitoes as dengue and chikungunya. ZIKV started circulating in Nicaragua in January 2016, in particular in Managua, were the catchment area of the study is located. Because the clinical presentation of the three diseases is very similar, the introduction CHIKV and ZIKV in the study population will likely increase the number of dengue-like illnesses in our cohort study and confound characterization of the epidemiology and clinical presentation of DENV infections. Thus, it is important to test for CHIKV and ZIKV infection in the cohort.

Re-enrollment and extension for children from 15 to 17 years of age
Dengue cases have decreased continually in the cohort. Recent statistical analyzes revealed a large force of infection in 1997-1999 and a gradual decline thereafter. A change in the rate of specific immunity to dengue virus (DENV) in children was also observed over time: in 2004, approx. 50% of children $\geq$5 years were DENV-positive, but in 2015, this level of seropositivity was only reached to children of 11 years. With respect to Zika it was found that Seroprevalence increased with age. Due to these new findings it is important to study dengue, chikungunya and Zika in older children. We therefore want to include children 15 to 17 years of age in the cohort study.

3. Collaborative Research

a) If any non-UCB institutions or individuals are engaged in the research, explain their human research roles and what human subjects training they have/PI has planned to provide.

This study will involve the following collaborating institutions. All Nicaraguan institutions are part of the Nicaraguan Ministry of Health and fall under IRB review conducted by the CNDR/MOH (see Section 3b):

Centro de Salud Sócrates Flores Vivas (HCSFV), Managua, is a Ministry of Health health center that serves as the primary site for the pediatric dengue cohort study and provides healthcare to the study participants. At the Health Center Dr. Guillermina Kuan, a licensed physician and head of the Health Center, will be responsible for overseeing participant care.

Center, will be responsible for overseeing participant care.

Hospital Infantil Manuel de Jesus Rivera (HIMJR), Managua, is the national pediatric reference hospital. It is the best-equipped pediatric facility in the country. All cases of severe dengue in the greater metropolitan area of Managua are referred to the HIMJR. All suspected dengue cases from the cohort presenting with signs of alarm are referred to study physicians at the HIMJR.

National Virology Laboratory, Centro Nacional de Diagnóstico y Referencia, Ministry of Health, Managua, performs all serological and virological tests for dengue diagnosis and will process the samples collected as part of this study. Dr. Angel Balmaseda is the Head of the National Virology Laboratory and the Major Foreign Collaborator (Foreign Prinicipal Investigator) of the site. Dr. Balmaseda is responsible for the study site in Nicaragua.

The Sustainable Sciences Institute (SSI) oversees the conduction of the study at the three institution mentioned above. SSI is a non-profit organization that has an agreement with the Nicaraguan Ministry of Health to administer collaborative research projects such as this study. SSI relies on the Nicaraguan Ministry of Health's IRB approval, as registered in the OHRP database (FWA00009658).

Dr. Aubree Gordon is an Assistant Professor in the Department of Epidemiology, University of Michigan, Ann Arbor. Dr. Gordon will analyze data generated by the study and contribute to design study instruments (e.g. questionnaires). Dr. Gordon specializes in the epidemiologic features of infectious diseases in tropical regions. She has been working with the Nicaraguan Ministry of Health and Dr. Harris for the past 10 years. She has completed CITI online human subjects training, and the NIH human subjects course. Dr. Gordon has taught a responsible conduct of research course, meeting NIH requirements, at UC Berkeley.

The following institutions are collaborating on investigations that do NOT involve human subjects research, but rather analysis of coded samples withOUT personal identifiers in order to meet the scientific objectives of the grants/study.

Dr. Mark Loeb at McMaster University in Canada has extensive experience in host genetic studies, and has just finished one contract with NIH examining host susceptibility or resistance to West Nile virus infection. We will collaborate with Dr. Loeb and a network he has put together in the Americas as well as Asia to examine host genetic contributions to severe disease as well as protection against dengue virus as part of a dengue host genetics contract from NIH that was recently awarded.

Dr. Perera, Rushika at the Colorado State University (CSU) is an expert in the field of RNA Virology. She will use the metabolomics platform at CSU to identify new biomarkers for dengue diagnosis and prognosis. This analysis will be performed using serum as well as non invasive samples such as urine and saliva.

Dr. Miriam Merad at the Icahn School of Medicine at Mount Sinai in New York is recognized for her studies on the mechanisms that regulate the development and function of innate myeloid cells including dendritic cells and macrophages. Dr. Sacha Gnjatic, also at the Icahn School of Medicine at Mount Sinai, is an expert in the identification of T cell epitopes and the mapping of T cell responses to specific antigens. Together, they will characterize immune signatures in dengue virus infections, including innate immune response and competence and T cell and dendritic cell function.

Dr. Eric Schadt at the Icahn School of Medicine at Mount Sinai in New York has published extensively on and pioneered research in integrating diverse types of large scale, high dimensional data to construct system-level, predictive models of complex phenotypes like common human diseases. Dr. Jun Zhu, also at

---

the Icahn School of Medicine at Mount Sinai is an expert in Bioinformatics. Together they will coordinate data analysis generated by various collaborators of the Dengue Human Immunology Project Consortium (DHIPC) and develop models to identify signatures that define immune response and categories/fingerprints/profiles that correlate with infection outcome.

Dr. Andrew Kasarskis at the Icahn School of Medicine at Mount Sinai in New York is an expert in large-scale biology and application of systems biology to biomedical research. His group will be in charge of data management and dissemination from/to collaborators of the Dengue Human Immunology Project Consortium (DHIPC).

Dr. John Tsang leads a laboratory focusing on systems and quantitative immunology at the National Institute of Allergy and Infectious Diseases (NIAID), National Institutes of Health (NIH). He also co-directs the Trans-NIH Center for Human Immunology (CHI) and leads its research program in systems human immunology. In relation to analysis of selected samples from the Nicaraguan cohort study, he will supervise the bioinformatic processing of the RNASeq or microarray gene expression data obtained. He will lead the design of and contribute substantially to the statistical analyses and quantitative modeling to investigate the effects of age, gender, and age-dependent and age-independent inter- vs. intra-individual variation in gene expression and immune profile in the study population. He will have access to limited metadata, as prepared by study data managers using un-linkable codes and de-identified data, and selected pre-collected de-identified specimens collected in PAXgene solution.

Dr. Popper is a Senior Research Scientist at Stanford University. The primary focus of his research has been to explore the use of the transcriptome as a tool for understanding the epidemiology of infectious disease and the biological basis for the variation in clinical outcomes. Dr. Popper will contribute to the overall study design and will work on functional and statistical analysis of processed RNAseq data from selected participants of the Nicaraguan cohort study to investigate the effects of age, gender, and individual variation in gene expression and immune profile in the study population. He will also coordinate efforts with Dr. Harris and researchers in Nicaragua to optimize subject and sample selection and associated metadata to generate hypotheses for future studies. He will have access to limited metadata, as prepared by study data managers using un-linkable codes and de-identified data, and selected pre-collected de-identified specimens collected in PAXgene solution.

b) If any non-UCB institutions or individuals are collaborating in the research, complete the table below and attach any relevant IRB approvals in the Attachments section.

Non-UCB institutions

Protocol Title: Pediatric Cohort Study of Dengue Transmission in Nicaragua
Protocol Type: Biomedical Non-Exempt
Date Submitted: Draft
Approval Period: Draft
Important Note: **This Print View may not reflect all comments and contingencies for approval. Please check the comments section of the online protocol.**
Questions that appear to not have been answered may not have been required for this submission. Please see the system application for more details.

Institution Name	Individual Contact/ Affiliate of Institution	FWA #	Local IRB Review? (Y or N)	IRB Approval Date	IRB Approval Expiration Date
Centro Nacional de Diagnostico y Referencia	Ministry of Health, Nicaragua	00009658	Y	03/26/2018	03/31/2019
Colorado State University	Rushika Perera	00000647	Y	04/11/2018	04/10/2019
University of Michigan, Ann Arbor	Aubree Gordon	00004969	Y	08/04/2017	08/03/2018
Icahn School of Medicine at Mount Sinai	Ana Fernandez-Sesma	00005656	Y	07/07/2017	06/16/2018
National Institutes of Healh	John Tsang		N		
Stanford University	Stephen Popper		N		

4. Qualifications of Study Personnel

a) **Explain expertise of Principal Investigator, Student/Postdoc Investigator, Faculty Sponsor (if applicable), any Co-Investigators or other key personnel listed in the application, and how it relates to their specific roles in the study team.**

Dr. Eva Harris is a Professor in the Division of Infectious Diseases and Vaccinology in the School of Public Health at UC Berkeley and the PI of this study, and she is a well-recognized expert in dengue and infectious diseases. She has collaborated on infectious disease research with the Nicaraguan Ministry of Health for the past 28 years, and with her Nicaraguan colleagues has been conducting studies of dengue in children in Nicaragua for the last 21 years. Dr. Harris has completed human subjects training offered through UC Irvine, NIH (Human Participant Protections Education for Reseach Teams), and the CITI online human subjects training course for Good Clinical Practices. Her CITI training certificate has been submitted to CPHS. She is fluent in written and spoken Spanish, and conducts all business and conversation with her Nicaraguan collaborators in Spanish.
Dr. Josefina Coloma is a Project Scientist in the Division of Infectious Diseases and Vaccinology in the School of Public Health at UC Berkeley and a Co-Investigator of this study. She has worked with Dr. Harris for over 15 years on dengue research and international scientific capacity building. Dr. Coloma has completed human subjects training offered through the NIH and CITI.

Dr. Raquel Burger-Calderon is a postdoctoral fellow in the Division of Infectious Diseases in the School of Public Health at UCB and one of the coordinators of this study. She worked on infectious diseases for 5 years while earning her doctoral degree in Microbiology and Immunology. She subsequently earned a MPH in Epidemiology before joining UCB. She has 7 years of experience with clinical studies and recently

---

MPH in Epidemiology before joining UCB. She has 7 years of experience with clinical studies and recently completed the UCB human subjects CITI training. Hence, she is well suited to implement the clinical and laboratory study objectives described here. Raquel Burger-Calderon will oversee local data collection, cleaning and analysis while she will have no direct contact with the subjects. Fausto Bustos will be conducting data analysis and working from UCB and he will have no direct contact with the subjects.

Anna Gajewski is a study coordinator stationed in Managua, Nicaragua. She earned her Master of Public Health (MPH, Epidemiology) from the Rollins School of Public Health, Emory University, Atlanta, GA in May of 2014. She has worked as program director and study coordinator in international public health organizations (Peru and Nicaragua) since 2011 and recently completed the UCB human subjects CITI training. Anna will help coordinate the study implementation, along with data cleaning and analysis while she will have no direct contact with the subjects.

b) In case of international research, describe the expertise you have, or have access to, which prepares you to conduct research in this location and/or with this subject population, including specific qualifications (e.g., relevant coursework, background, experience, training). Also, explain your knowledge of local community attitudes and cultural norms, and cultural sensitivities necessary to carry out the research. See Human Subjects Research in an International Setting and <a href='http://cphs.berkeley.edu/international.pdf' target='_blank'>CPHS Guidelines on Research in an International Setting.

Dr. Harris has considerable expertise building scientific capacity in public health and conducting infectious disease research in developing countries. Dr. Harris has collaborated continuously with the Nicaraguan Ministry of Health (MOH) for over 28 years, with 21 years of research and capacity development focused on strengthening dengue diagnosis and research. All of Dr. Harris' research and projects in Nicaragua fall under a Memorandum of Understanding with the MOH, and she meets regularly with the Minster of Health and other MOH officials to discuss new research directions and current projects. In 1998, Dr. Harris and her Nicaraguan colleagues began a hospital-based study at HIMJR on risk factors for severe dengue, and since 2004 she has run a long-term, community-based cohort study of dengue in the Americas in a health center in Managua and another community-based project on dengue control and prevention. The research staff in this study and others run by Dr. Harris is 95% Nicaraguan. Local study coordinators and staff are responsible for all aspects of contact with human subjects, including development of culturally appropriate materials and protocols. Dr. Harris is fluent in written and spoken Spanish, and conducts all business and conversation with Nicaraguan collaborators in Spanish.

Dr. Coloma has collaborated on infectious disease research with ministries of health in several Latin American countries. In Nicaragua, she has participated in several research studies with Dr Harris as Co investigator and Co-PI for 14 years. Dr Coloma is a project scientist in Dr. Harris laboratory and has extensive experience in Project management. She is a native Spanish speaker.

Dr. Burger-Calderon is a trained microbiologist and epidemiologist and accumulated 7 years of experience in translational research. Her multicultural background, being Swiss with Colombian ancestry and having been trained in the US for the past 10 years, sets her up well to accomplish the established research goals in Nicaragua while assuring that NIH and CDC general and human subject guidelines are met. She is fluent in Spanish and English and writes well in both languages. Furthermore, her Latino background allows here to strengthen the culturally appropriate research materials and protocols in the Nicaraguan context. She is based in Managua, Nicaragua, acting as a study coordinator and researcher.

Protocol Title: Pediatric Cohort Study of Dengue Transmission in Nicaragua

Protocol Type: Biomedical Non-Exempt

Date Submitted: Draft

Approval Period: Draft

Important Note: This Print View may not reflect all comments and contingencies for approval. Please check the comments section of the online protocol.
Questions that appear to not have been answered may not have been required for this submission. Please see the system application for more details.

Protocol Title: Pediatric Cohort Study of Dengue Transmission in Nicaragua
Protocol Type: Biomedical Non-Exempt
Date Submitted: Draft
Approval Period: Draft
Important Note: **This Print View may not reflect all comments and contingencies for approval. Please check the comments section of the online protocol.
Questions that appear to not have been answered may not have been required for this submission. Please see the system application for more details.**

---

* * * Subject Population * * *

5. Subject Population

a) Describe proposed subject population, stating age range, gender, race, ethnicity, language and literacy.

All participants will live in District II, Managua, Nicaragua, the catchment area for the Health Center Socrates Flores Vivas (HCSFV). No discrimination will take place in terms of race or gender; however, the age of study participants will be limited to children ages 2 to 17 years old as children have the highest burden of dengue. The subjects may be of three vulnerable populations: the illiterate, the poor, and children.

b) State the maximum number of subjects planned for the study. This number should account for all subjects to be recruited, including those who may drop out or be found ineligible. Explain how number of subjects needed to answer the research question was determined.

Since the beginning of the study in 2004 there have been a total 9,138 study participants. The current cohort size is 3,806 participants aged 2-14 years. The study maintains ~200-300 children in each 1-year age group from 2-14 years. Approximately 300 participants between 15 and 17 years of age will be included in the cohort this year due the age expansion, however these participants have been part of the cohort in the past and therefore do not increase the overall study participant number.

To maintain the cohort age structure and adjust for participants who age out of the cohort study, we plan to enroll up to 320 2-year old annually. Moreover, based on our study data from August 2004 to June 2010, we anticipate an annual loss to follow-up of ~6%. Thus, to maintain the cohort size and compensate for loss to follow-up, we plan to enroll up to 300 children aged 3 to 14.

We estimate that this sample size should yield 270-1155 infections per year, assuming an annual incidence of DENV infection of 7-30% (based on prior observed annual incidence rates in this and neighboring districts in Managua since 2001). Estimating that 10-20% of the infections will be symptomatic; this would result in 27-231 symptomatic dengue cases. Of these, ~15% (4-35 children) would require hospitalization for dengue, although we expect 2- to 3-fold more hospitalizations due to other diseases with similar clinical presentations. This cohort size ensures that even in years of relatively low transmission, a minimum number of symptomatic cases will be obtained.

The project period of our extended IRIDA/NIH funds (titled Epidemiological, Clinical & Immunological Studies of Zika, dengue and chikungunya, Grant Number: 5R01AI099631-07) is from 06/28/2012 to 05/31/2022. Hence, we plan to continue enrolling ~620 participants annually (up to 320 2-year old to maintain the cohort age structure and up to 300 participants aged 3 to 14 to compensate loss to follow-up) for ~5 years (2018-2022), thus increasing study sample size from 10,600 to 12,200. Further, NIH approval has been received to use the funds for an extended cohort, now incl. children 15 to 17 yrs of age (see attachments in section 22 of the protocol).

c) If any proposed subjects are children/minors, prisoners, pregnant women, those with physical or cognitive impairments, or others who are considered vulnerable to coercion or undue influence, state rationale for their involvement.

The subjects may be of three vulnerable populations: the illiterate, the poor, and children. This study consists entirely of children, as children bear the greatest burden of dengue in Nicaragua. The purpose of this study is to maintain a pediatric cohort to determine the transmission of the disease and its clinical manifestations so therefore the population must be children. This study will also include the poor and illiterate as they represent a part of the general population in Managua and have a high burden of dengue disease.

6. Recruitment

a) Explain how, where, when, and by whom prospective subjects will be identified/selected and approached for study participation. If researcher is subject's instructor, physician, or job supervisor, or if vulnerable subject groups will be recruited, explain what precautions will be taken to minimize potential coercion or undue influence to participate. See CPHS Guidelines on Recruitment for more information.

Due to the changes made to the letter of consent and the age extension up to 17 years of age, the whole cohort will be re-consented before starting the next annual 2019 sampling. The age extension will be offered to all 14 year old children who are participating in the study at the moment and the children who participated in the study before but who were retired in 2018 because they turned 15 years old. These re-consents and extensions will be offered upon visits to the health center or by household visits. Note that 15 years olds who actively withdrew from the study will not be contacted for re-enrollment, consistent with the protocol and the original consent form to which they agreed.

Following rationale will be presented when re-contacting former participants (those who have aged-out at age 15 years old) to participants/parents as to why they are being re-contacted and re-invited to participate in the study up to 17 years, which is consistent with the parent permission and assent forms: Based on the ongoing study it seems as if dengue, Zika and chikungunya affect older children more than younger children. We would like to determine whether that is true and understand why in order to improve medical care for children in the future.

New participants will be enrolled mainly during the annual sampling between February and April of each year and if necessary throughout the year to maintain the age structure of the cohort. Recruitment of new children will occur annually through house-to-house visits in the CSSFV service area or in the CSSFV through promotion of the study to the parents of 2-year-old who attend the Health Center for medical consultation. Appropriately trained study staff will provide information to parents or guardians, one-on-one or in small groups and answer questions related to the study. The discussion will begin with a preliminary explanation of the study, and the consent letter will be reviewed and read aloud together, providing a more detailed explanation of all aspects of the study.

b) Describe any recruitment materials (e.g., letters, flyers, advertisements [note type of media/where posted], scripts for verbal recruitment, etc.) and letter of permission/cooperation from institutions, agencies or organizations where off-site subject recruitment will take place (e.g., another UC campus, clinic, school district). Attach these documents in Attachments section. Please see eProtocol Attachments Check List for Non-Exempt Applications for more information.

No recruitment materials are used.

c) Will anyone who will be recruiting or enrolling human subjects for this research receive compensation for

---

each subject enrolled into this protocol? If yes, please identify the individual(s) and the amount of payment (per subject and total).

No, payment to all staff is done on a monthly basis and is not dependent on the number of individuals enrolled in the study.

7. Screening

a) **Provide criteria for subject inclusion and exclusion. If any inclusion/exclusion criteria are based on gender, race, or ethnicity, explain rationale for restrictions.**

Inclusion Criteria:
To participate in the study: 1) informed consent must be obtained from the child's parent or legal guardian, 2) children aged 6 and over must give verbal assent to participate, 3) children in rented households must have lived in the neighborhood for at least 2 years and children in owner-occupied households must have lived in the neighborhood for at least 6 months, 4) families must intend to remain in the neighborhood for the length of the study, 5) participants must have a vaccination card in order to confirm age and residence, or, if a vaccination card cannot be presented, participants must have a pre-existing medical chart at the HCSFV, and 6) if a child is sick (fever and/or chronic illness), he/she must be examined at the HCSFV to determine eligibility for participation in the cohort.

Exclusion Criteria:
Children will be excluded from the study if: 1) their parent or guardian fails to give informed consent, 2) they decline to give verbal assent if aged 6 and over, 3) they do not meet minimum residency requirements, 4) they have a chronic illness that puts them at risk for serious health complications, 5) their parents are not willing to bring them to the HCSFV for their medical attention during the study period, or 6) they do not meet or are unable to adequately document meeting the appropriate age of enrollment.

b) **If prospective subjects will be screened via tests, interviews, etc., prior to entry into the "main" study, explain how, where, when, and by whom screening will be done. NOTE: Consent must be obtained for screening procedures as well as "main" study procedures. As appropriate, either: 1) create a separate "Screening Consent Form;" or 2) include screening information within the consent form for the main study.**

No screening tests will be conducted.

8. Compensation and Costs

a)

Describe plan for compensation of subjects. If no compensation will be provided, this should be stated. If subjects will be compensated for their participation, explain in detail about the amount and methods/ terms of payment.

Include any provisions for partial payment if subject withdraws before study is complete.

When subjects are required to provide Social Security Number in order to be paid, this data must be collected separately from consent documentation. If applicable, describe security measures that will be used to protect subject confidentiality.

If non-monetary compensation (e.g., course credit, services) will be offered, explain how

All study participants will be given school supplies or the equivalent valued at ~US$1.50. This gift is used instead of monetary compensation in order to avoid undue influence to participate, as this is a vulnerable population. The study gift will be given to the parent each year after the blood draw.

b) **Discuss reasoning behind amount/method/terms of compensation, including appropriateness of compensation for the study population and avoiding undue influence to participate.**

Compensation in the form of small gifts appropriate for this population has been chosen in order to avoid undue influence of monetary compensation in the study population, which is largely poor.

c) **Costs to Subjects. If applicable, describe any costs/charges which subjects or their insurance carriers will be expected to pay. (If there are no costs to subjects or their insurers, this should be stated.)**

There is no cost to subject or insurers for participating in this study.

Protocol Title: Pediatric Cohort Study of Dengue Transmission in Nicaragua
Protocol Type: Biomedical Non-Exempt
Date Submitted: Draft
Approval Period: Draft
Important Note: **This Print View may not reflect all comments and contingencies for approval. Please check the comments section of the online protocol.**
Questions that appear to not have been answered may not have been required for this submission. Please see the system application for more details.

---

* * * Study Procedures, Alternatives to Participation * * *

9. Study Procedures

a) Describe in chronological order of events how the research will be conducted, providing information about all study procedures (e.g., all interventions/interactions with subjects, data collection procedures etc.), including follow-up procedures. If any interviews, questionnaires, surveys, or focus groups will be conducted for the study, explain and attach one copy each of all study instruments (standard and/or non-standard) in the Attachments section. Please see eProtocol Attachments Check List for Non-Exempt Applications for more information. If the proposed research involves use of existing data/specimens, describe how data/specimens will be acquired.

Eligibility for the study is determined by the study criteria, after which the informed consent process will take place. These steps will occur in either of the following ways: 1) at the time of the child's routine medical visit to the HCSFV or 2) through a home visit. Demographic data and information regarding each participant's health history will be obtained at the time of enrollment and should take approximately 15 minutes. The house of each participant will be geo-referenced using satellite technology.

Annual Sample Collection

Each year between February and April, a study nurse will make contact with the participant and parent to collect a sample of blood from each participant. This contact will either occur at the health center or during a visit to the participant's home. If the drawn blood volume will depend on the age of the child. If the child is between 2 and 14 years of age a 8 ml blood sample will be collected at this time. If the child is 15 years of age or older a 14 ml blood sample will be drawn. The nurse will also ask questions about the participant's health, including whether the participant has been ill, has attended the HCSFV, or has traveled out of Managua. The entire appointment will last from 20 minutes to an hour depending on the number of children who are present. The total time commitment for the study will range between 1-3 hours per year, which will range widely depending on participant-driven medical visits and associated symptoms.

Within at least a week but no later than a month of the participant's annual sample collection, study participants will be expected to attend the HCSFV to (a) receive results from the Complete Blood Count (CBC) test performed on the blood sample, (b) make and receive a photo ID card, if the participant does not already have one, (c) scan the participant's fingerprint to facilitate future registration and identification, if such a scan is not already on file. A physician will be available to explain the laboratory results and make a follow-up appointment if necessary. This process generally takes 15 minutes or less per child.

Continuous Procedures over the Duration of the Study

Attending the HCSFV for illness
All children who report to the HCSFV will be channeled through the reception mechanism at the health center's Admission desk. All children will be asked if they are participants in the study, and if so will be asked to provide a means for rapid identification with either their study ID card or a fingerprint scan, both of which are linked to a study code by which participant's medical charts are organized. The parent and child will then be directed to the clinic for consultation with a physician (available 24h/day for ambulatory care). A standard form will be completed for all medical consults.

Suspected dengue cases

Suspected dengue cases

The study aims to obtain clinical information and isolate the virus from participants with dengue. As dengue has a wide range of clinical manifestations, especially in children (where not all symptoms may be reported), the criteria used will be broader than the MOH definition of a clinical case of dengue. If a patient is suspected of dengue or a fever of unknown origin (FUO), an acute phase serum sample will be obtained, as is routinely done by the MOH for suspected dengue cases. Any virus obtained will be stored for future studies. Specifically, MOH guidelines specify collection of 5ml of blood on day 1 of illness for laboratory analysis for diagnosis of dengue, and ~ 1ml of blood daily for (CBC) clinical management, as ordered by the physician. The remainder will be used for research purposes as part of this study; thus, no additional blood is drawn from suspected dengue cases during the acute phase than that mandated by MOH norms. It is difficult to specify the exact amount remaining since it depends on how much serum is obtained from the sample after separation from blood cells and how is needed for the different diagnostic assays, which can vary depending on whether repetitions are necessary. A 5-ml convalescent sample will be collected 14-21 days post-illness onset as part of this study because it is necessary for serological diagnosis of dengue (levels of anti-DENV antibodies in paired acute- and convalescent-phase samples are analyzed in parallel), although it is not part of the MOH norms for primary care. Additionally, non-invasive samples will be collected from suspected dengue cases on the initial visit to the Health Center. Up to 10 ml of saliva will be collected specifically for this study, aliquotted and stored. As urine is already collected as part of the MOH guidelines at the Health Center for patients suspected of dengue, no additional urine collection will be necessary. Up to 10 ml of urine will be separated from the rest of the sample and refrigerated until further processing and storage.
Moreover, suspected dengue cases (or a subset of those) will be screened for DENV infection by rapid test and/or RT-PCR. A second 5-ml blood sample will be collected on day 4-6 post-illness from participants with confirmed dengue. As a rule, we will draw the blood together with the daily CBC sampling whenever possible in order to minimize discomfort and risk for the participant. Please refer to the attachment “Blood Collection during the Study” for a summary of blood collection over the duration of the study, and a comparison of MOH norms and study procedures.

Additionally, during periods when Chikungunya virus (CHIKV) and Zika virus (ZIKV) circulate, suspected dengue cases will be tested for CHIKV and ZIKV (by RT-PCR, serology, and/or other diagnostic test) using the acute and/or convalescent samples collected for DENV testing.

For clinical management of suspected dengue cases, the Nicaraguan MOH traditionally collects a 5ml acute sample for serological testing and surveillance purposes, as well as daily blood samples of 1ml for CBC analysis with platelet count during the first week of symptoms, as prescribed by the physician, as mentioned above. This allows for appropriate management of dengue cases, since falling platelet counts (leading to thrombocytopenia, or <100,000 platelets/mm3) and rising hematocrit (to >20% of baseline value) are signs of the potentially fatal DHF/DSS. Platelet counts and hematocrit determination are routinely performed by all MOH centers whenever possible during the acute phase of the illness. Daily clinical and laboratory review will continue until the fever and altered laboratory results subside, the child is transferred to the hospital, or a cause of illness other than dengue is confirmed. If the suspected dengue/FUO case requires hospitalization, the study will arrange for transfer from the HCSFV to the national pediatric reference Hospital Infantil Manuel de Jesus Rivera (“La Mascota”), where the usual criteria for hospitalization will be applied to the study participant. The child will be accompanied by study personnel to the hospital until one of the study doctors at the hospital checks him/her. The study will cover the costs of the extra laboratory tests that will help the hospital doctors treat the child. These tests will be performed daily and will include an X-ray or sonogram (for detection of pleural effusion, a sign of plasma leakage) and collection of 5ml of blood, as per MOH and hospital norms. Again, the remainder will be used

for study purposes. Blood is normally taken daily when children are in the hospital for dengue, so this does not entail additional blood draws.

Confirmed Zika cases

Additional samples will be collected from participants with a laboratory-confirmed ZIKV infection, a second 5-ml blood sample will be collected on day 4-6 post-illness from these participants, as for DENV-positive participants (see above). As a rule, we will draw the blood together with a CBC sampling whenever possible in order to minimize discomfort and risk for the participant. This sample will be used to study the immune response against ZIKV infections. Moreover, up to 10 ml of saliva and up to 10 ml of urine will be collected, aliquotted and stored on day 4-6 post-illness and in the convalescent phase of the disease (approximately 14-21 days post-illness onset). These samples will be used to develop and/or validate new, non-invasive diagnostic tests for ZIKV.

If a ZIKV-positive participant develops neurological symptoms, the study will arrange for transfer from the HCSFV to the national pediatric reference Hospital Infantil Manuel de Jesus Rivera ("La Mascota"), where the participant will be assessed by a study neurologist. If the participant requires hospitalization for his/her neurological symptoms, the study neurologist will evaluate the patient during the course of hospitalization, as needed in accordance with the patient's neurological signs and symptoms. The results of the neurological assessment and of the hospitalization follow-up, as well as the results of any specific testing conducted, will be recorded in the participant's clinical file. This information will be accessible to the physicians providing care to the participant at the HCSFV and will be used for research purposes.

Screening of cases of fever with a known focus (diagnosis other than dengue or undifferentiated fever)

To ensure that the HCSFV is successfully diagnosing febrile illnesses and screening for dengue, a percentage of cases of fever with an identified focus will be screened for dengue. During the period of high dengue transmission, permission will be requested from parents to collect an acute sample for dengue testing from children who have blood taken for CBC by doctor's orders (totaling 3ml), as well as from children with diagnoses of respiratory infections or urinary tract infections (UTI), justified as "Búsqueda Activa" ("active search") as directed by the MOH (without requiring additional consent). This is expected to yield acute blood specimens from an average of 60% of cases with a differential diagnosis. During the rest of the year, permission will be requested from parents to collect an acute sample (totaling 3 ml) for dengue testing from children who have blood taken for CBC by doctor's order (an average of ~25%).

Other contact from the study and HCSFV

To evaluate compliance with the study procedure of early presentation to the HCSFV when ill and to promote Health Center attendance, study nurses will visit the houses of study participants. A full-scale undertaking where over 80% of houses are visited will occur at least once per year to evaluate participant compliance and satisfaction. At these visits, a brief questionnaire will be administered regarding the child's health and attendance to the HCSFV over the previous several months.

Additional Informed Consent Processes

An informed consent process that requests permission to store clinical information, and blood samples for future studies will occur (Consent Form Part B) at the time of enrollment, as well as an additional informed consent process that requests permission to store and use DNA from new participants (Consent Form Part C). No additional samples will be taken to obtain the DNA.

For Consent part D:
Parents/guardians of participants who are 14 years of age will be asked to review an additional informed consent process to allow for the collection and use of samples up to 17 years of age (Consent Form Part D). The same will be done for parents/guardians of participants who turned 15 years of age in 2018 and were removed from the cohort. It will be carried out along with the other aspects of re-consent (Consent Form Parts A to C). Parents/guardians of participants will not be re-consented if their child was withdrawn by the parents/actively withdrew from the study.

For assent script part D:
Verbal assent will be completed for participants 14 and 15 years of age. Participants who are close to reaching the age of 15, will be asked to undergo a process of assent, for the realization of samples for diagnosis of dengue, chikungunya and Zika, as described in the main part of the study. Retired participants who have already reached the age of 15 will be offered the extension to 17 years with an assent process, as described in the main part of the study. A consent form will be read to the participants, and their consent obtained prior to taking the samples.

Please note that the description of the quantity of blood, which is often stated in terms of teaspoons and tablespoons in the United States, is perceived in Nicaragua as confusing, with the potential of having the participant think that they might be consuming something. The use of “ml or cc” to describe the volume is more culturally acceptable. It is a term understood by lay people, as it is common for people to use liquid medicines for injections.

Procedures External to Direct Patient Participation

Procedures pertaining to viral RNA and blood samples
After serum/plasma from the blood specimens have been separated for serological, virological, and blood chemistry testing, the remainder will be separated to collect PBMCs and aliquoted and stored at -70°C or liquid nitrogen until further processing. Any virus isolated will be stored as first-passage aliquots at -70°C and liquid nitrogen for future use in pathogenesis studies. Dengue virus (DENV) RNA spanning the entire length of the viral genome will be amplified from acute phase sera, a subset of PBMCs, and isolated viruses. Sera and PBMCs will be stored in aliquots at -70°C and liquid nitrogen, respectively, for use in evaluation and validation of new diagnostic tests (such as RVPs, NS1 capture ELISAs, and type- and epitope-specific assays), measurement of neutralizing and enhancing anti-DENV antibodies and DENV-specific serum avidity, evaluation of the host immune response (e.g., microarray and proteomic studies, B and T cell responses ex vivo), and additional and as yet undefined investigations. The sample collected on day 4-6 of illness will be used to study acute-phase immunological responses; in particular cell-mediated immunity. A small aliquot of the blood sample (0.8ml) will be stored in Trizol or the equivalent for further RNA extraction and gene expression analysis.

Procedures pertaining to participant DNA
For patients and their parents/guardians who consent to genetic studies, DNA will be preserved in its crude cellular form until time of extraction. The DNA samples will be retained by the primary investigators (project coordinators) and stored at the University of California, Berkeley, or in the CNDR in Managua, Nicaragua. DNA samples will be used for investigation of genomic single nucleotide polymorphisms (SNPs), HLA loci, and killer immunoglobulin receptor (KIR) polymorphisms in relation to susceptibility/resistance to severe dengue in future collaborative genetic studies.

DNA amplified from dengue viral RNA, DENV RNA, and crude or extracted human genomic DNA will be

---

sent from Nicaragua to collaborators via UC Berkeley (see list of sites below). This is for processing but not for long term storage. Clinical information in databases that contain NO personal identifiers will be sent to collaborators to accompany the samples. All host nucleic acids sent to collaborators will be returned to UC Berkeley or destroyed after genetic analysis is completed.

Procedures pertaining to saliva and urine samples
Urine and saliva samples will be refrigerated immediately after collection. Then they will be aliquotted and stored at -70°C. Coded samples with all personal identifiers unlinked will be sent to Colorado State University for metabolomics analysis.

Sample storage
Long-term storage will only occur at the University of California, Berkeley, and the CNDR in Managua Nicaragua. As mentioned in Section 3a, the following collaborating institutions will participate in the analysis and processing of coded samples without personal identifiers in order to meet the scientific objectives of the grants/study:

The Broad Institute in Cambridge, Massachusetts; Integral Molecular in Philadelphia, Pennsylvania; Dr. David Relman's Laboratory at Stanford University School of Medicine; the DeRisi Laboratory in the Department of Biochemistry, University of California, San Francisco; Dr. Munir Alam's Laboratory in the Human Vaccine Institute at Duke University Medical Center; Dr. James Crowe's Laboratory in the Department of Pediatrics, Microbiology and Immunology at the Vanderbilt University Medical Center, Dr. Andrew Fire's Laboratory at Stanford University; Dr. Philip Armstrong at the Connecticut Agricultural Experiment Station; Dr. Laura Kramer, Director of the Arbovirus Laboratories at the Wadsworth Center at the New York State Dept Health in Albany, NY; Dr. Theodore Pierson at the Laboratory for Infectious Diseases at the NIH; Dr. Wei-Kung Wang's laboratory in the Department of Tropical Medicine, Medical Microbiology and Pharmacology in the John A. Burns School of Medicine at the University of Hawaii at Manoa; Dr. Mark Loeb at McMaster University in Canada; Dr. Michael Bamshad's laboratory at the University of Washington (Seattle) and Dr. Barry Beaty at Colorado State University. DNA may be analyzed at the Broad Institute and in Dr. Bamshad and/or Dr. Loeb's laboratory.

b) Explain who will conduct the procedures, where and when they will take place. Indicate frequency and duration of visits/sessions, as well as total time commitment for the study.

Contact with study participants will be primarily through personnel (physicians, nurses, and reception area staff) of the HCSFV. Study nurses will perform the house visits. House visits will be performed as described above for annual sample collection and to collect data on study attendance. Participants will generally receive no more than 2 house visits per year. The exception to this will be when a home visit becomes necessary for medical follow-up. Home visits generally are of 15 minutes or less duration, and the process of drawing blood should take approximately 5 minutes per child. Study participants developing neurological symptoms, including weakness of the limbs, numbness and tingling sensations, will be transferred to the national pediatric reference Hospital Infantil Manuel de Jesus Rivera ("La Mascota"). A study neurologist will perform necessary neurological tests and monitor the child closely during hospitalization.The total time commitment for the study will range between 1-3 hours per year, excluding medical care which will range widely depending on participant-driven medical visits and associated symptoms.

c) Identify any research procedures that are experimental/investigational. Experimental or investigational

---

procedures are treatments or interventions that do not conform to commonly accepted clinical or research practice as may occur in medical, psychological, or educational settings. Note: if the study only involves standard research or clinical procedures, state "N/A."

No procedures are experimental or investigational.

d) If a placebo will be used, provide rationale and explain why active control is not appropriate.

No placebo will be used.

e) If any type of deception or incomplete disclosure will be used, explain what it will entail, why it is justified, and what the plans are to debrief subjects. See CPHS Guidelines on Deception and Incomplete Disclosure for more information. Any debriefing materials should be included in the Attachments section.

No deception or lack of full disclosure will be used.

f) State if audio or video recording will occur and for what purpose (e.g. transcription, coding facial expressions).

No audio or video taping will occur.

10. Alternatives to Participation

Describe appropriate alternative resources, procedures, courses of treatment, if any, that are available to prospective subjects. If there are no appropriate alternatives to study participation, this should be stated. If the study does not involve treatment/intervention, enter "N/A" here.

N/A

Protocol Title: Pediatric Cohort Study of Dengue Transmission in Nicaragua
Protocol Type: Biomedical Non-Exempt
Date Submitted: Draft
Approval Period: Draft
Important Note: This Print View may not reflect all comments and contingencies for approval. Please check the comments section of the online protocol.
Questions that appear to not have been answered may not have been required for this submission. Please see the system application for more details.

---

* * * Radiation * * *

11. Ionizing Radiation (e.g. X-ray) and Non-ionizing Radiation (e.g. MRI)

a) Do you intend to use ionizing radioactive materials or ionizing radiation-producing devices in your research (e.g., injectable, oral, x-rays, etc.)? CAUTION: The UCB Radioactive Materials License does not permit human research using radioactive materials or radiation from such materials. N

If Yes, provide Radiation Use Authorization (RUA) number(s):

Note: The research may not proceed without an RUA. Please visit:
http://www.ehs.berkeley.edu/how-create-new-radiation-use-authorization-rua

b) Do you intend to use any non-ionizing radiation sources (laser or magnetic sources) in your research? N

If Yes, provide Laser Use Registration (LUR) number(s):

And/or Magnetic Inventory number(s):

Note: The research may not proceed without an LUR or Magnetic Inventory Number. Please visit:
http://ehs.berkeley.edu/laser-safety/how-do-i-register-my-new-laser

c) Describe the source of ionizing radiation or non-ionizing radiation.

N/A

Protocol Title: Pediatric Cohort Study of Dengue Transmission in Nicaragua
Protocol Type: Biomedical Non-Exempt
Date Submitted: Draft
Approval Period: Draft
Important Note: This Print View may not reflect all comments and contingencies for approval. Please check the comments section of the online protocol.
Questions that appear to not have been answered may not have been required for this submission. Please see the system application for more details.

---

* * * Medical Equipment, Investigational Devices * * *

12. Medical Equipment

If the research involves use of medical equipment, explain whether the equipment is approved for marketing and routinely employed in clinical practice.

Physicians and medical staff will use standard medical equipment such as blood pressure cuffs, thermometers, stethoscopes, etc.

13. Investigational Devices

List in the table below all Investigational Devices to be used on subjects

Investigational Devices

---

* * * Drugs, Reagents, or Chemicals * * *

14. Drugs, Reagents, or Chemicals

a) List in the table below all investigational drugs, reagents or chemicals to be administered to subjects during this study.

b) List in the table below all commercial drugs, reagents or chemicals to be administered to subjects during this study.

Protocol Title: Pediatric Cohort Study of Dengue Transmission in Nicaragua
Protocol Type: Biomedical Non-Exempt
Date Submitted: Draft
Approval Period: Draft
Important Note: This Print View may not reflect all comments and contingencies for approval. Please check the comments section of the online protocol.
Questions that appear to not have been answered may not have been required for this submission. Please see the system application for more details.

---

* * * Risks and Discomforts * * *

15. Risks and Discomforts

a) **Describe all known risks and discomforts associated with study procedures, whether physical, psychological, economic or social (e.g., pain, stress, invasion of privacy, breach of confidentiality), noting the likelihood and degree of potential harm.**

The risks associated with blood draws include potential complications such as pain, bleeding and bruising at the venipuncture site; in very rare instances, infection may occur. There is no anticipated risk for the collection of urine and saliva. There is minimal risk that patient confidentiality could be violated. The repercussions of this risk are minimal since there is no social stigma associated with dengue virus infection. There is no anticipated legal risk associated with the study.

b) **Discuss measures that will be taken to minimize risks and discomforts to subjects. In terms of minimizing a confidentiality breach, simply refer to section 17 (Confidentiality).**

To minimize blood draw risks, all blood draws will be venous collections performed by professional health care personnel. The use of stringent aseptic technique and post-venipuncture pressure to the site should minimize bleeding and infectious complications. No risk is anticipated for the collection of urine and saliva. Nevertheless, urine and saliva collection will be performed by trained study personnel and will follow international and HCSFV guidelines. All information obtained from the subjects will be confidential. To ensure this, all samples will be labeled with a study code, and clinical information and test results will be kept in databases without personal identifiers. The separate password-guarded database linking the study code to personal identifiers will only be accessible to study coordinators and key personnel. With these measures in place, it is unlikely that confidentiality will be breached.

c) **If applicable, indicate if a particular study treatment or procedure may involve risks to the subject (or to the embryo or fetus, if the subject is or may become pregnant) that are currently unforeseeable.**

N/A

d) **If applicable, describe the Data Safety Monitoring Plan (DSMP). NIH may require a DSMP for some projects.**

N/A

e) **Explain how unanticipated negative outcomes/experiences or serious adverse events will be managed. (NOTE: This may apply in social-behavioral as well as biomedical research, e.g., undue stress or anxiety of subject, breach of confidentiality via loss of laptop computer with study data. Provisions should be made and described here if applicable.)**

Severe disease or death is a potential outcome of dengue virus infection, especially if a child presents to the heath center late in the course of DHF or DSS. As this is a community-based study, the probability of

---

the heath center late in the course of DHF or DSS. As this is a community-based study, the probability of a death occurring in study participants is low, and multiple measures have been put into effect to encourage participants to present in the first few days of fever to minimize this risk. All participants will receive the best medical care available in Nicaragua. If a non-serious adverse event, such as a local infection, occurs as a result of a blood draw or any other study procedure, the participant will be treated at the HCSFV or if necessary at the HIMJR without any charge to the participant. The study will provide reimbursement (50 cordobas) per trip for any necessary medical visits. Any compensation for the adverse event will follow the University of California policy. If a serious adverse event occurs, the study investigators are familiar with the University of California policy and will follow the policy.

f) Discuss plans for reporting unanticipated problems involving risks to subjects or others, or serious adverse events, to CPHS. (This applies to all types of research.) See Adverse Event and Unanticipated Problem Reporting.

In the event of any adverse event related to study participation, the study personal will notify both CPHS and the Nicaraguan IRB. This notification may initially come through phone communication within 7 days, but in all cases a written report will be filed in the native language and to CPHS within 14 days of the adverse event. If there is a breach of confidentiality via unauthorized computer access or the loss of a computer, we will notify CPHS within the above noted timeframes and the Nicaraguan IRB immediately.

g) Describe plans for provision of treatment for study-related injuries, and how costs of injury treatment will be covered. If the study involves more than minimal risk, indicate that the researchers are familiar with and will follow University of California policy in this regard, and will use recommended wording on any consent forms (see CPHS Informed Consent Guidelines).

If a non-serious adverse event occurs as a result of any study procedure, the participant will be treated at the HCSFV without any charge to the participant. The study will provide reimbursement (50 cordobas) per trip for any necessary medical visits. Any compensation for the adverse event will follow the UC policy.

---

* * * Benefits, Confidentiality * * *

16. Benefits

Describe any potential benefits to the individual subject, group of subjects, and/or society. If subjects will not benefit directly from study procedures, this should be stated.

NOTE: Do not include compensation/payment of subjects in this section, as remuneration is not considered a "benefit" of participation in research.

The benefits to subjects for participation in this study are as follows:
1.The study participant will contribute to the research effort to improve control, prevention and knowledge of dengue.
2.The study participant will receive educational information about dengue and its prevention and control.
3.The study participant will receive annual results of a complete blood chemistry, including platelets, that can identify anemia and other illnesses.
4.The study participant will have access to study physicians 24 hours a day, 7 days a week at the HCSFV regardless of the cause of illness.
5.The study participant will have access to transportation 24 hours a day, 7 days a week from the health center to other centers of medical attention in cases of emergency.
6.The study participant will have access to timely laboratory results as study personnel at the health center's clinical laboratory is available 24 hours a day, 7 days a week. In other health centers in Managua, clinical laboratories are only open during working hours.

17. Confidentiality and Privacy

NOTE: See CPHS Data Security Policy and Guidelines before completing this section.

a) **If reviewing or accessing Protected Health Information (PHI) from UC Berkeley's Tang Center, Optometry Clinic, Psychology Clinic, Intercollegiate Athletics, or Human Resources for activities preparatory to research, describe the process and confirm that the health information will not be removed from the "covered entity".**

N/A

b) **What identifiable participant data will you obtain? Note: Audio, photo, and video recordings are generally considered identifiable unless distinguishing features can be successfully masked.**

Individually identifiable information will be collected from participants, including names, parents' names, birth dates, addresses, and GPS coordinates of the household. These data will be stored on computer and will be accessible to study personnel at the HCSFV on a per-need basis. Such persons include data entry personnel, admissions personnel, and persons supervising and managing study data. All computers with

---

participants' data will be password-protected (see below). The participants’ code will be accessible to study personnel who may provide medical attention to the participant, and who are shown the study ID card.
No individual identities will be used in any reports or publications resulting from this study.

c) If obtaining existing data/specimens, will you have access to identifiers? Please see The Industry Alliance Office website for requirements when receiving existing data/specimens for research.

Yes.

d) Explain how the confidentiality of subject information will be maintained. Include:

i. Who will have access to study records/specimens? If the study is subject to FDA regulations, include a statement that the FDA might inspect the records of the study.

Physicians, nurses, and HCSFV staff who work on the study will have access to personal identifiable information on clinical records and charts as necessary for providing medical care. Other than these clinical staff, only the Data Managers, Site and Study Coordinators and PI will have access to identifiable information. While participant names will appear on clinical forms, which are source materials and part of the participant's medical records, names and other identifiable information will not be present in study databases where clinical, laboratory and demographic information collected from the patient will be stored for research purposes. The GPS data will be used to produce maps for use in the field. All such maps will be collected at the end of all field-work and stored in a locked file cabinet. Laboratory technicians at the CNDR, will have access to the specimens. Tubes and vials will be labeled with a barcode sticker, such that clinical specimens sent to the CNDR for processing and storage will not carry patient names or identifiable information.
This study is not subject to FDA regulations.

ii. How the records will be secured (e.g., password-protected computer, encrypted files, locked cabinet). Response should be consistent with CPHS Data Security Policy.

At the time of enrollment, contact information for the participants will be obtained by an interviewer and entered into a password-protected database of identifiable information and kept in a locked office.
Each study participant will have a medical chart at the HCSFV. They will be accessed by medical personnel and limited study personnel, and will be kept in a locked room per Ministry of Health norms. Surveys and consent forms are kept separately in a locked file cabinet in a locked office.
Each subject will have a unique identification code, and all specimens will be labeled only with this code. The key to this code will be kept in the password-guarded master study database. The study coordinators and key personnel will be the only ones with access to the master database. Access to the database containing identifiable information will be strictly controlled. All devices and computers with identifiable data will be password-protected. Databases with identifiable data will also be password-protected. All passwords will meet the CPHS definition of a secure password (i.e. passwords of 10 characters or more which contain at least one of each of the following: upper-case letter, lower-case letter, number and symbol)
Secure data encryption will be used whenever identifiable information is: 1) stored in a networked computer/device; 2) stored in mobile devices (smart phones, laptops, tablet computers) which are

computer/device; 2) stored in mobile devices (smart phones, laptops, tablet computers) which are not permanently stored in a secure location; and 3) transmitted via email or internet. No identifiable information will be stored in the cloud. All encryption passwords will meet the CPHS definition of a secure password.
Encoded databases, which do not contain identifiable information, will be created and used for all analyses. Collaborating research groups and institutions will be sent coded samples with all personal identifiers unlinked; this includes any researchers performing genetic studies. No collaborating research group or institution will have access to the database which relates the study code to personal identifying information.

iii. **How long study data will be retained, including signed consent forms. Data retention specifications should adhere to the regulatory requirements applicable to the study (e.g. DHHS, OCR [HIPAA], FDA, etc.).**

As a part of enrollment, participants will be asked for permission to store samples and use samples/data in other studies. Samples/data will be kept for those who granted permission for storage and future use. Data collection instruments are included as a part of the child's medical record. Therefore, the data collection instruments will not be destroyed by study staff. Moreover, as per UCOP data retention policies, all data on participants will be stored for at least 7 years after they reach age of maturity (18 years of age).

iv. **When audio/video recordings will be transcribed and when they will be destroyed (if ever).**

N/A

e) **Identifiers should be removed from data/specimens as soon as possible following collection, except in cases where the identifiers are embedded (e.g., voices in audio or faces in video recordings). If data are coded in order to retain a link between the data and identifiable information, explain where the key to the code will be stored, how it will be protected, who will have access to it, and when it will be destroyed.**

Specimens are marked only with the study code. The key to this code will be kept in an encrypted and password-guarded master study database. The study coordinators and key personnel will be the only ones with access to the master database. As a part of enrollment, participants will be asked for permission to use samples/data in other studies. The link between data and identifiable information will be destroyed 7 years following study completion for all those who did not grant permission for future use. The link between data and identifiable information will be destroyed 15 years following study completion for all those who did grant permission for future use (signed Part B).

f) **Describe how identifiable data will be transferred (e.g., courier, mail) or transmitted (e.g., file transfer software, file sharing, email). If transmitted via electronic networks, describe how you will secure the data while in transit (e.g., prior encryption). If not applicable, enter N/A.**

Identifiable data may be transmitted via email or internet. In these cases, secure data encryption is used. All encryption passwords meet the CPHS definition of a secure password. No identifiable information is stored in the cloud.

---

g) Will subjects be asked to give permission for release of identifiable data (e.g., for future studies, publications, presentations, etc.), now or in the future? If so, explain here and include appropriate statements in the consent materials. See Media Records Release Form template for guidance.

No.

h) Explain how subject privacy will be protected (e.g., conducting interviews in a discreet location).

Appropriate measures will be taken to protect subject's privacy. Medical consults will be conducted in dedicated rooms in the study health center, only accessible to the participant, his/her parent/guardian, and medical personnel. Interviews (at enrollment and during annual sample collection) will be conducted in the waiting area of the health center or at the participant's home. In the waiting area of the health center, interviews will be conducted as discretely as possible (in close contact with the interviewer).

Protocol Title: Pediatric Cohort Study of Dengue Transmission in Nicaragua
Protocol Type: Biomedical Non-Exempt
Date Submitted: Draft
Approval Period: Draft
Important Note: **This Print View may not reflect all comments and contingencies for approval. Please check the comments section of the online protocol.**
Questions that appear to not have been answered may not have been required for this submission. Please see the system application for more details.

---

* * * Potential Financial Conflict of Interest * * *

18. Potential Financial Conflict of Interest

Individuals who have independent roles in projects and who are responsible for the design, analysis, conduct, or reporting of the results of research performed (or to be performed) under a human subjects protocol must disclose whether or not they have a financial interest in or association with the sponsor or the company supplying materials, drugs, or devices for the project. This checklist pertains to the entire project team working under the protocol. Any individual who has a conflict must comply with University regulations and procedures for disclosure of financial conflict of interest.

See Conflict of Interest Committee Website for more information.

Please answer the following questions:

Does any member of the project team (defined as UCB or non-UCB personnel working under the protocol) with substantive responsibility for the design, conduct, or reporting of activities under the protocol, or any member of their immediate family (defined as spouse, dependent child or registered domestic partner) have any of the following relationships with the non-UC entity financing the research to be done under the protocol or the non-UC entity supplying materials, drugs or devices being tested under the protocol:

1. N **Positions of management (e.g., board member, scientific advisor, director, officer, partner, trustee, employee, consultant).**
2. N **Equity interest (e.g., stock, stock options, investment, or other ownership).**
3. N **Rights to a pending patent application or issued patent to any invention(s), or license rights or copyright for software that has a direct relationship to the project proposed.**

If the answer to any of the above is Yes, then each individual with any "Yes" response (s) must submit a Human Subjects Financial Conflict of Interest Form and include it in the Attachments section of the protocol.

NOTE: When review by the COI Committee is required, CPHS approval of protocols will be contingent upon the disclosure and resolution of all financial conflicts of interest, as determined by the COI Committee.

---

* * * Informed Consent * * *

19. Informed Consent

Add the consent documents and/or waivers needed for this research using the table at the bottom of the page, including any translated versions. For any translated consent, include an affirmation of the translation's accuracy, indicating who is affirming the accuracy (PI, Co-PI, or Student Investigator), in the Consent/Waiver Description or in the Attachment section. Describe the consent process and provide justification for any waivers for each consent document, translation, and/or waiver. The various consent/waiver options are described below.

Note: DO NOT include child assent documents, parent permission documents or waivers here (these are addressed in the next section).

Altered and Unsigned Consent - A consent document that has omitted required information and does not include a place for a participant's signature. This means that CPHS is being asked to waive one or more elements of consent in addition to the requirement for documented consent.

Altered Consent Form - A consent form that has omitted required information. This means that the CPHS is asked to waive one or more required elements of informed consent. For example, if the purpose of the study will not be disclosed to participants in order to avoid bias, this option should be selected because disclosure of the "purpose" is a required element of informed consent. The form must include a signature line and date line for the individual to sign if he or she agrees to participate.

Consent Form - A standard consent document that embodies all of the required information (elements of informed consent) designed to help an individual make an informed decision about whether or not to participate in the research. The form must include a signature line and date line for the individual to sign if he or she agrees to participate. The Consent Form can also be presented as a "short form" document stating that the required elements of informed consent have been presented orally to the participant. When the short form method is used, a "summary" of the information that is presented to the participant must also be provided for CPHS approval and there must be an impartial witness to the oral presentation. The witness must sign the summary as well as the short form and the participant must sign the summary. The "short form" method may be used in circumstances where oral presentation of consent is preferable or necessary, e.g., subjects are illiterate in English or their native language.

Consent Waiver - No consent will be sought at all. This means that the CPHS is asked to waive the requirement for informed consent. This option is often appropriate for research that involves use of existing data or samples

Unsigned Consent - A document that embodies all of the required information (elements of informed consent), but does not include a place for a participant to indicate with a signature that he or she agrees to

take part in the research. This means that the CPHS is asked to waive the requirement for documented (signed) consent. For example, if consent will be obtained verbally or using a button on the web, this option should be selected.

•Informed Consent Guidelines, Templates and Sample Forms

•Informed Consent Policies and Procedures

•Consent Builder: Online Tool for Creating Consent Forms

Informed Consent

Consent/Waiver Description	Consent Type	Consent Document

Informed Consent

Consent/Waiver Description (e.g. Consent for Group A, Waiver for Group B, Surrogate Consent for Group C) — Waiver - all forms in Section 20

Consent Type — Consent Waiver

For the CPHS to approve a waiver of one or more elements of informed consent, either criterion A or B must be met. Select the applicable criterion and provide justification in the box below.

A. (1) The research involves no more than minimal risk of harm to the subjects;

(2) The waiver or alteration will not adversely affect the rights and welfare of the subjects;

(3) The research could not practicably be carried out without the waiver or alteration; and

(4) Whenever appropriate, the subjects will be provided with pertinent information after participation.

Y **B.** (1) The research or demonstration project is to be conducted by or subject to the approval of state or local officials and is designed to study, evaluate, or otherwise examine: (i) public benefit or service programs; (ii) procedures for obtaining benefits or service; (iii) possible changes in or alternatives to those programs or procedures; or (iv) possible changes in methods or levels of payment for benefits or services under those programs;and

(2) The research could not practicably be carried out without the waiver or alteration.

A waiver was marked because all consent forms require parental permission, and are thus uploaded under Section 20.

---

* * * Child Assent & Parent Permission * * *

20. Child Assent and Parent/Guardian Permission

Add each child assent document, parent/guardian permission document, and/or waiver needed for this research using the table at the bottom of the page, including any translated versions. For any translated consent, include an affirmation of the translation's accuracy, indicating who is affirming the accuracy (PI, Co-PI, or Student Investigator), in the Consent/Waiver Description or in the Attachment section. Describe the consent process and provide justification for any waivers for each consent document, translation, and/or waiver. The various consent/waiver options are described below.

Altered and Unsigned Parent/Guardian Permission Form - A parent permission document that has omitted required information (elements) and does not include a place for a parent to indicate with a signature that he or she agrees to permit the child's participation. This means that CPHS is being asked to waive one or more elements of consent in addition to the requirement for documented consent.

Altered Parent/Guardian Permission Form - A permission form that has omitted required information (elements). This means that the CPHS is asked to waive one or more required elements of informed consent. However, the form must include signature and date lines for the parent(s)/guardian(s) to sign if the child is permitted to take part in the research.

Assent Document - A form or script of the information that will be conveyed to the child about the study. In general, researchers must obtain the affirmative agreement of children ages seven years and older for their participation. Assent forms should be written at a level understandable to the child. If the study includes a broad age range of children, more than one assent form may be needed (i.e., an assent form suitable for a 15 year old is not usually suitable for a 7 year old child).

Assent Waiver - No child assent will be sought at all. This means that CPHS is asked to waive the requirement for child assent. Among other circumstances, this option is appropriate when the capability of the child to understand the research is too limited or when the research holds out a prospect of direct benefit that is important to the health or well being of the child.

Parent/Guardian Permission Form - A document that embodies all of the required information (elements of informed consent) designed to help the parent/guardian of a child make an informed decision about whether or not to permit the child's participation in the research. The form must include signature and date lines for the parent(s)/guardian(s) to sign if the child is permitted to take part in the research.

Permission Waiver - No parent/guardian permission will be sought at all. This means that the CPHS is asked to waive the requirement for parent/guardian permission. This option, for example, is often appropriate for research designed to study conditions in children or a study population for which parental permission is not a reasonable requirement to protect the children (e.g., neglected or abused children).

Unsigned Parent/Guardian Permission - A parent permission document that embodies all of the required information (elements of informed consent), but does not include a place for a parent to indicate with a signature that he or she agrees to permit the child's participation. This means that the CPHS is asked to waive the requirement for documented (signed) consent.

•Child Assent and Parent Permission Guidelines, Templates, and Sample Forms

•Policies and Procedures on Child Assent and Parent Permission

Documents and Waivers

Permission/Assent Description	Assent or Permission Type	Assent/Permission Document

Documents and Waivers

Permission/Assent Description (e.g. Assent for Group A, Permission for Group A, Waiver of Parent Permission for Group B, Assent for Group B etc) — Consent Form New Participants - Spanish

Assent or Permission Type — Parent/Guardian Permission Form

Attach Assent or Permission documents (in Word format) — X | Assent/Permission Document | Dengue_consent_long_Span_v12.0

Explain how, where, when, and by whom the permission of the parent or legal guardian of the child will be obtained.

See description provided under English version. The PI and Student PI both affirm the translation's accuracy.

If any special circumstances are involved, address considerations appropriately.

Permission/Assent Description (e.g. Assent for Group A, Permission for Group A, Waiver of Parent Permission for Group B, Assent for Group B etc) — Assent Script - Spanish

Assent or Permission Type — Assent Document

Attach Assent or Permission documents (in Word format) — X | Assent/Permission Document | Dengue_assentscript_Span_v5.0

Explain how, where, when, and by whom assent will be obtained from the minor/child.

Protocol Title: Pediatric Cohort Study of Dengue Transmission in Nicaragua
Protocol Type: Biomedical Non-Exempt
Date Submitted: Draft
Approval Period: Draft
Important Note: **This Print View may not reflect all comments and contingencies for approval. Please check the comments section of the online protocol.**
Questions that appear to not have been answered may not have been required for this submission. Please see the system application for more details.

Study personnel will introduce the study and recruit participants. One-on-one or in small groups, information regarding the study will be provided to the adult(s) living in the same household as the case or in close proximity. The discussion will begin with a preliminary explanation of the study, and the assent form will be reviewed together, providing a more detailed explanation of all of the aspects of the study. Only participants and children whose parents have consented to their participation and children over age 6 who have given assent will be permitted to enter the study. Among children, assent will be sought from subjects over 6 years of age, and training for informed assent will include details on only obtaining guardian consent from the appropriate person for participants under 18 years of age. The parental permission will be explained verbally, and a document, written in Spanish, will be presented to the potential participant. Additional consent processes will be conducted for the option to allow future use (Part B) and use of DNA for study related purposes (Part C). The interviewer will also sign the consent form as a witness/study representative. An unsigned copy of the consent form will serve as an information sheet describing the study procedures, risks, benefits, and contact information, and will be given to the study participant. Verbal assent will be obtained from all children aged 6 and older following parental consent. The informed consent and assent processes will involve reading the consent document (see attachment)aloud to all potential participants; those who are not able to write will be asked to sign their name with their fingerprint.

The PI and Student PI both affirm the translation's accuracy.

Describe any additional/appropriate measures that will be in place to protect this vulnerable population, if any.

Permission/Assent Description (e.g. Assent for Group A, Permission for Group A, Waiver of Parent Permission for Group B, Assent for Group B etc)	Consent Form New Participants - English		
Assent or Permission Type	Parent/Guardian Permission Form		
Attach Assent or Permission documents (in Word format)	X	Assent/Permission Document	Dengue_consent_long_Eng_v12.0

Explain how, where, when, and by whom the permission of the parent or legal guardian of the child will be obtained.

**Protocol Title:**	Pediatric Cohort Study of Dengue Transmission in Nicaragua
**Protocol Type:**	Biomedical Non-Exempt
**Date Submitted:**	**Draft**
**Approval Period:**	**Draft**
**Important Note:**	**This Print View may not reflect all comments and contingencies for approval. Please check the comments section of the online protocol. Questions that appear to not have been answered may not have been required for this submission. Please see the system application for more details.**

This consent form will be used to consent new participants. Study personnel will introduce the study and recruit participants. One-on-one or in small groups, information regarding the study will be provided to the adult(s) living in the same household as the case or in close proximity. The discussion will begin with a preliminary explanation of the study, and the consent form will be reviewed together, providing a more detailed explanation of all of the aspects of the study. Only participants and children whose parents have consented to their participation and children over age 6 who have given assent will be permitted to enter the study. Among children, assent will be sought from subjects over 6 years of age, and training for informed consent will include details on only obtaining guardian consent from the appropriate person for participants under 18 years of age.

The consent will be explained verbally, and a document, written in Spanish, will be presented to the potential participant. Additional consent processes will be conducted for the option to allow use of DNA for study related purposes. The interviewer will also sign the consent form as a witness/study representative. An unsigned copy of the consent form will serve as an information sheet describing the study procedures, risks, benefits, and contact information, and will be given to the study participant. Verbal assent will be obtained from all children aged 6 and older following parental consent.

The informed consent and assent processes will involve reading the consent document (see attachment)aloud to all potential participants; those who are not able to write will be asked to sign their name with their fingerprint. Some subjects may belong to lower socio-economic classes. As there are no financial incentives associated with this study, we do not think that this study involves any coercion; therefore, we do not feel we are putting this population at greater risk. Training for informed consent will include details on only obtaining guardian consent from the appropriate person.

If any special circumstances are involved, address considerations appropriately.

Permission/Assent Description (e.g. Assent for Group A, Permission for Group A, Waiver of Parent Permission for Group B, Assent for Group B etc)	Assent Script - English		
Assent or Permission Type	Assent Document		
Attach Assent or Permission documents (in Word format)	X	Assent/Permission Document	Dengue_assentscript_Eng_v5.0

Explain how, where, when, and by whom assent will be obtained from the minor/child.

Study personnel will introduce the study and recruit participants. One-on-one or in small groups, information regarding the study will be provided to the adult(s) living in the same household as the case or in close proximity. The discussion will begin with a preliminary explanation of the study, and the assent form will be reviewed together, providing a more detailed explanation of all of the aspects of the study. Only participants and children whose parents have consented to their participation and children over age 6 who have given assent will be permitted to enter the study. Among children, assent will be sought from subjects over 6 years of age, and training for informed assent will include details on only obtaining guardian consent from the appropriate person for participants under 18 years of age. The parental permission will be explained verbally, and a document, written in Spanish, will be presented to the potential participant. Additional consent processes will be conducted for the option to allow future use (Part B) and use of DNA for study related purposes (Part C). The interviewer will also sign the consent form as a witness/study representative. An unsigned copy of the consent form will serve as an information sheet describing the study procedures, risks, benefits, and contact information, and will be given to the study participant. Verbal assent will be obtained from all children aged 6 and older following parental consent. The informed consent and assent processes will involve reading the consent document (see attachment)aloud to all potential participants; those who are not able to write will be asked to sign their name with their fingerprint.

Describe any additional/appropriate measures that will be in place to protect this vulnerable population, if any.

Some subjects may belong to lower socio-economic classes. As there are no financial incentives associated with this study, we do not think that this study involves any coercion; therefore, we do not feel we are putting this population at greater risk. Training for informed consent will include details on only obtaining guardian consent from the appropriate person.

Permission/Assent Description (e.g. Assent for Group A, Permission for Group A, Waiver of Parent Permission for Group B, Assent for Group B etc)	Parent Permission Form Part D - English		
Assent or Permission Type	Parent/Guardian Permission Form		
Attach Assent or Permission documents (in Word format)	X	Assent/Permission Document	Dengue_permission_Part_D_Expand_Edad_Eng_v1.0

Explain how, where, when, and by whom the permission of the parent or legal guardian of the child will be obtained.

This consent form Part D will be used to (i) expand the study age of current 14 year old participants to 17 years of age and (ii) re-consent participants who have reached 15 years of age in 2018 to join the cohort again until they are 17 year of age.

Study personnel will explain why we are expanding the study time or re-consenting and information regarding the general study will be provided to the adult(s) and children living in the same household, either one-on-one or in small groups.

The informed consent processes will involve reading the consent document (see attachment) aloud and the document, written in Spanish, will be presented to the potential participant. The interviewer will also sign the consent form as a witness/study representative. An unsigned copy of the consent form will serve as an information sheet describing the study procedures, risks, benefits, and contact information. Those who are not able to write will be asked to sign their name with their fingerprint. Some subjects may belong to lower socio-economic classes. As there are no financial incentives associated with this study, we do not think that this study involves any coercion; therefore, we do not feel we are putting this population at greater risk. Training for informed consent will include details on only obtaining assent and guardian consent from the appropriate person.

Only participants and children whose parents have consented to their participation and children over age 6 who have given assent will be permitted to expand or re-enter the study. Verbal assent will be obtained from all children aged 6 and older following parental consent. Informed guardian consent will be obtained from the appropriate person for participants under 18 years of age.

If any special circumstances are involved, address considerations appropriately.

Permission/Assent Description (e.g. Assent for Group A, Permission for Group A, Waiver of Parent Permission for Group B, Assent for Group B etc)	Parent Permission Form Part D - Spanish		
Assent or Permission Type	Parent/Guardian Permission Form		
Attach Assent or Permission documents (in Word format)	X	Assent/Permission Document	Dengue_permission_Part_D_Expand_Edad_Span_v1.0

Explain how, where, when, and by whom the permission of the parent or legal guardian of the child will be obtained.

**Protocol Title:**	Pediatric Cohort Study of Dengue Transmission in Nicaragua
**Protocol Type:**	Biomedical Non-Exempt
**Date Submitted:**	**Draft**
**Approval Period:**	**Draft**
**Important Note:**	**This Print View may not reflect all comments and contingencies for approval. Please check the comments section of the online protocol.**<br>**Questions that appear to not have been answered may not have been required for this submission. Please see the system application for more details.**

This consent form Part D will be used to (i) expand the study age of current 14 year old participants to 17 years of age and (ii) re-consent participants who have reached 15 years of age in 2018 to join the cohort again until they are 17 year of age.

Study personnel will explain why we are expanding the study time or re-consenting and information regarding the general study will be provided to the adult(s) and children living in the same household, either one-on-one or in small groups.

The informed consent processes will involve reading the consent document (see attachment) aloud and the document, written in Spanish, will be presented to the potential participant. The interviewer will also sign the consent form as a witness/study representative. An unsigned copy of the consent form will serve as an information sheet describing the study procedures, risks, benefits, and contact information. Those who are not able to write will be asked to sign their name with their fingerprint. Some subjects may belong to lower socio-economic classes. As there are no financial incentives associated with this study, we do not think that this study involves any coercion; therefore, we do not feel we are putting this population at greater risk. Training for informed consent will include details on only obtaining assent and guardian consent from the appropriate person.

Only participants and children whose parents have consented to their participation and children over age 6 who have given assent will be permitted to expand or re-enter the study. Verbal assent will be obtained from all children aged 6 and older following parental consent. Informed guardian consent will be obtained from the appropriate person for participants under 18 years of age.

The PI and the Student PI both affirm the translation's accuracy.

If any special circumstances are involved, address considerations appropriately.

Permission/Assent Description (e.g. Assent for Group A, Permission for Group A, Waiver of Parent Permission for Group B, Assent for Group B etc)	Assent Script Part D - English		
Assent or Permission Type	Assent Document		
Attach Assent or Permission documents (in Word format)	X	Assent/Permission Document	Dengue_assentscript_Part_D_Eng_v1.0 (2)

Explain how, where, when, and by whom assent will be obtained from the minor/child.

This assent script associated with form Part D will be used to (i) expand the study age of current 14 year old participants to 17 years of age and (ii) re-consent participants who have reached 15 years of age in 2018 to join the cohort again until they are 17 year of age.

Study personnel will explain why we are expanding the study time or re-consenting and information regarding the general study will be provided to children/families living in the same household, either one-on-one or in small groups.

Verbal assent will be obtained from all children aged 6 and older following parental consent. The verbal assent processes will involve reading the document (see attachment) aloud and any questions will be answered.

Some subjects may belong to lower socio-economic classes. As there are no financial incentives associated with this study, we do not think that this study involves any coercion; therefore, we do not feel we are putting this population at greater risk. Training for informed consent will include details on only obtaining assent and guardian consent from the appropriate person.

Only participants and children whose parents have consented to their participation and children over age 6 who have given assent will be permitted to expand or re-enter the study.

Describe any additional/appropriate measures that will be in place to protect this vulnerable population, if any.

Permission/Assent Description (e.g. Assent for Group A, Permission for Group A, Waiver of Parent Permission for Group B, Assent for Group B etc)	Assent Script Part D - Spanish		
Assent or Permission Type	Assent Document		
Attach Assent or Permission documents (in Word format)	X	Assent/Permission Document	Dengue_assentscript_Part_D_Span_v1.0

Explain how, where, when, and by whom assent will be obtained from the minor/child.

Protocol Title: Pediatric Cohort Study of Dengue Transmission in Nicaragua

Protocol Type: Biomedical Non-Exempt

Date Submitted: Draft

Approval Period: Draft

Important Note: **This Print View may not reflect all comments and contingencies for approval. Please check the comments section of the online protocol.**
Questions that appear to not have been answered may not have been required for this submission. Please see the system application for more details.

This assent script associated with form Part D will be used to (i) expand the study age of current 14 year old participants to 17 years of age and (ii) re-consent participants who have reached 15 years of age in 2018 to join the cohort again until they are 17 year of age.

Study personnel will explain why we are expanding the study time or re-consenting and information regarding the general
study will be provided to children/families living in the same household, either one-on-one or in small groups.

Verbal assent will be obtained from all children aged 6 and older following parental consent. The verbal assent processes will involve reading the document (see attachment) aloud and any questions will be answered.

Some subjects may belong to lower socio-economic classes. As there are no financial incentives associated with this study, we do not think that this study involves any coercion; therefore, we do not feel we are putting this population at greater risk. Training for informed consent will include details on only obtaining assent and guardian consent from the appropriate person.

Only participants and children whose parents have consented to their participation and children over age 6 who have given assent will be permitted to expand or re-enter the study.

The PI and Student PI both affirm the translation's accuracy.

Describe any additional/appropriate measures that will be in place to protect this vulnerable population, if any.

---

* * * HIPAA * * *

21. Health Insurance Portability and Accountability Act (HIPAA)

The HIPAA Privacy Rule establishes the right of an individual to authorize a covered entity, such as a health plan, health care clearinghouse or health care provider, to use and disclose his/her Protected Health Information (PHI) for research purposes. UC Berkeley's covered entities are the University Health Services (including its health care services on behalf of Intercollegiate Athletics) and the Optometry Clinic. The Privacy Rule defines the elements of individual information that comprise PHI and establishes the conditions under which PHI may be used or disclosed by covered entities for research purposes. It also includes provisions to allow an individual's PHI to be disclosed or used in research without their authorization (i.e., IRB waiver of authorization). For more information, see CPHS Guidelines HIPAA and Human Subjects Research.

a. Does the study involve use of Protected Health Information (PHI) from a "covered entity" outside of UC Berkeley (i.e. another organization or institution)? For more information, see HIPAA and Human Subjects Research.

N

If Yes, explain what arrangements have been made to comply with the HIPAA requirements of the entity from which the PHI will be obtained:

b. Does the study involve use of a "Limited Data Set" from a covered entity? For more information, see HIPAA and Human Subjects Research Please see The Industry Alliance Office website for limited data set requirements.

N

If Yes, patient authorization for use of the data set is not required; however, you must have a data use agreement in place with the data holder from which the data will be obtained as required by HIPAA. Contact the Industry Alliance Office for further information at (510) 642-5766.

c. Does the study involve use of Protected Health Information (PHI) from UC Berkeley's University Health Services (including its health care services on behalf of Intercollegiate Athletics) and/or the Optometry Clinic?

N

If Yes (and a limited data set will not be used), EITHER request/add a Waiver/Alteration of HIPAA Authorization below OR provide a HIPAA Authorization Form in the Attachments section of the protocol.

Protocol Title: Pediatric Cohort Study of Dengue Transmission in Nicaragua

Protocol Type: Biomedical Non-Exempt

Date Submitted: **Draft**

Approval Period: **Draft**

Important Note: **This Print View may not reflect all comments and contingencies for approval. Please check the comments section of the online protocol.**
Questions that appear to not have been answered may not have been required for this submission. Please see the system application for more details.

HIPAA WAIVER/ALTERATION: For each waiver or alteration of the requirement for authorization from the patient for use of his or her PHI, provide justification in the table below.

Note: Use table below ONLY when requesting waiver/alteration of HIPAA authorization for use of PHI from UC Berkeley's Tang Health Center, the Human Resources Health Plan, Athletics and Recreational Sports, and/or the Optometry Clinic. For more information, see HIPAA and Human Subjects Research.
