# Supplementary material for: Prior dengue virus infection and risk of Zika: A pediatric cohort in Nicaragua
Source: PLoS Med. 2019 Jan 22;16(1):e1002726. doi: 10.1371/journal.pmed.1002726 (PMC6342296; doi:10.1371/journal.pmed.1002726)
Supplement: S2 Appendix — (DOCX) [file pmed.1002726.s002.docx]

STROBE Statement—Checklist of items that should be included in reports of ***cohort studies***

|  | Item No | Recommendation |
| --- | --- | --- |
| **Title and abstract** | 1 | 1. Indicate the study’s design with a commonly used term in the title or the abstract   Abstract states cohort study. |
|  |  | 1. Provide in the abstract an informative and balanced summary of what was done and what was found   Done. |
| Introduction | | |
| Background/rationale | 2 | Explain the scientific background and rationale for the investigation being reported  ZIKV shares extensive homology with dengue virus (DENV). The four DENV serotypes interact immunologically: infection with one serotype provides transient cross-protection against infection with heterologous serotypes, but sequential infection with different DENV serotypes is the most important risk factor for severe dengue disease, an effect that is mediated in part by antibody-dependent enhancement (ADE) [13-15]. In *in vitro* and in murine models, both cross-neutralization and enhancement between DENV and ZIKV have been observed [16-19]. However, experiments in rhesus macaques [20] and viral load and cytokine analysis in humans [21] do not support ZIKV enhancement by pre-existing DENV immunity. Interestingly, throughout the Americas, a precipitous decrease in the number of dengue cases was observed following widespread Zika epidemics [22], suggesting that ZIKV infection might induce cross-protective immune responses against DENV. However, the characterization of potential cross-protection between DENV and ZIKV requires knowledge of longitudinal pre-infection immune histories, which is only available in prospective cohort studies [23] (Introduction, paragraph 3) |
| Objectives | 3 | State specific objectives, including any prespecified hypotheses  Here, we describe the introduction of ZIKV into the Pediatric Dengue Cohort Study (PDCS), a long-standing pediatric dengue cohort established in 2004 in Managua, Nicaragua, in which the DENV immune history of the participants is well-characterized [24-26]. The incidence of symptomatic and inapparent ZIKV infections in 2016 was estimated, together with associated demographic risk factors. The effect of previous DENV infection on ZIKV infection and disease was also analyzed. (Introduction, paragraph 4) |
| Methods | | |
| Study design | 4 | The PDCS is an ongoing study of dengue (since August 2004), chikungunya (since September 2014), and Zika (since July 2015). Study design, population and detailed methods have been described previously [25-27]. Briefly, the PDCS is a community-based prospective study consisting of approximately 3,700 children uniformly distributed over each year of age between 2 and 14 years. The study was sized to examine the effects of repeat DENV infection. The study is based at a primary health center, the Health Center Sócrates Flores Vivas (HCSFV), in District II of Managua, the capital of Nicaragua. The study area consists of 17 neighborhoods with most inhabitants living at low- to mid-socioeconomic status. Primary health care is provided by study personnel to all participants, and acute illnesses are screened using the study case definition (see below). Initial recruitment into the study occurred through door-to-door visits. All children 2 to 9 years old living within the study area were invited to participate; the age range was then extended to 14 years old. Every March, healthy blood samples (annual samples) are collected, and additional participants are enrolled to maintain the cohort age structure and compensate for loss-to-follow-up. Children aged 2 years are also enrolled year-round to maintain the age structure. (Methods, paragraph 2) |
| Setting | 5 | Describe the setting, locations, and relevant dates, including periods of recruitment, exposure, follow-up, and data collection  The study is based at a primary health center, the Health Center Sócrates Flores Vivas (HCSFV), in District II of Managua, the capital of Nicaragua. The study area consists of 17 neighborhoods with most inhabitants living at low- to mid-socioeconomic status. (Methods, paragraph 2)  Symptomatic and inapparent DENV infections were recorded in the PDCS since study inception in August 2004 through a combination of RT-PCR, virus isolation and serological methods for symptomatic cases, and dengue iELISA on paired annual samples for inapparent infections [25, 26]. (Methods, paragraph 5)  A total of 3,893 children aged 2-14 years participated in the PDCS between January 1, 2016, and February 28, 2017. (Results, paragraph 2) |
| Participants | 6 | (*a*) Give the eligibility criteria, and the sources and methods of selection of participants. Describe methods of follow-up  Initial recruitment into the study occurred through door-to-door visits. All children 2 to 9 years old living within the study area were invited to participate; the age range was then extended to 14 years old. Every March, healthy blood samples (annual samples) are collected, and additional participants are enrolled to maintain the cohort age structure and compensate for loss-to-follow-up. Children aged 2 years are also enrolled year-round to maintain the age structure. (Lines 116-121)  Primary health care is provided by study personnel to all participants, and acute illnesses are screened using the study case definition (see below). (Methods, paragraph 2) |
|  |  | (*b*) For matched studies, give matching criteria and number of exposed and unexposed  N/A |
| Variables | 7 | Clearly define all outcomes, exposures, predictors, potential confounders, and effect modifiers. Give diagnostic criteria, if applicable  Symptomatic and inapparent DENV infections were recorded in the PDCS since study inception in August 2004 through a combination of RT-PCR, virus isolation and serological methods for symptomatic cases, and dengue iELISA on paired annual samples for inapparent infections [25, 26]. Infecting DENV serotype information is available for most symptomatic cases but only for a subset of the inapparent infections [37]. Prior DENV infection was defined as at least one inapparent or symptomatic infection since the participant entered the PDCS until the 2015/2016 season. Recent DENV infection was defined as an inapparent or symptomatic infection during the 2015/2016 season. Children with either a documented prior DENV infection or who entered the cohort DENV naïve and had no documented DENV infections were considered to have known DENV infection histories. (Methods, paragraph 5) |
| Data sources/ measurement | 8* | For each variable of interest, give sources of data and details of methods of assessment (measurement). Describe comparability of assessment methods if there is more than one group  A urine sample was collected from participants meeting these case definitions and presenting Monday to Friday, 7am to 5pm. RNA was extracted from acute serum and, as available, urine samples (QIAamp Viral RNA Mini Kit, Qiagen) and tested by real-time RT-PCR (rRT-PCR) for ZIKV, DENV and CHIKV RNA. Testing was performed in multiplex for the three viral RNAs [31, 32] or separately using a singleplex ZIKV assay [6] and a multiplex DENV/CHIKV assay [31]. Paired acute and convalescent samples were tested using in-house Zika and dengue IgM antibody capture enzyme-linked immunosorbent assays (MAC-ELISA) and Zika and dengue Inhibition ELISAs (iELISA) [15, 26, 33]. Additionally, pre- and post-infection (i.e., 2016 and 2017) annual samples were analyzed using a ZIKV NS1 blockade-of-binding (BOB) test [33, 34]. An algorithm was developed to integrate qualitative results from the two MAC-ELISAs and the ZIKV NS1 BOB assay, as well as quantitative results for iELISAs (see Classification Algorithm below). Cases meeting the testing definition were considered Zika cases (i.e., symptomatic ZIKV infections) if: 1) ZIKV RNA was detected by rRT-PCR in serum and/or urine, and/or 2) serological tests results were consistent with a ZIKV infection using the algorithm developed. Participants whose paired annual samples (i.e., 2016 and 2017) showed a ZIKV NS1 BOB ELISA seroconversion, but were not identified as a Zika case, were considered to have experienced an inapparent ZIKV infection. ZIKV infection refers to all identified ZIKV infections, whether a Zika case or an inapparent ZIKV infection. (Methods, paragraph 3) |
| Bias | 9 | Describe any efforts to address potential sources of bias  Analyses of the effect of DENV immunity were limited to children with known DENV infection histories. In addition, all ZIKV cases or infections that occurred prior to March 2016 (n=68) were excluded because: 1) The testing definition for Zika changed in February 2016, and 2) due to the potential DENV/ZIKV serological cross-reactivity in the ZIKV NS1 BOB assay when performed in samples collected early after the acute infection. (Methods, paragraph 6) |
| Study size | 10 | Explain how the study size was arrived at  The PDCS is a community-based prospective study consisting of approximately 3,700 children uniformly distributed over each year of age between 2 and 14 years. The study was sized to examine the effects of repeat DENV infection. (Methods, paragraph 2) |
| Quantitative variables | 11 | Explain how quantitative variables were handled in the analyses. If applicable, describe which groupings were chosen and why  N/A |
| Statistical methods | 12 | 1. Describe all statistical methods, including those used to control for confounding |
|  |  | (*b*) Describe any methods used to examine subgroups and interactions |
|  |  | (*c*) Explain how missing data were addressed |
|  |  | (*d*) If applicable, explain how loss to follow-up was addressed |
|  |  | (*e*) Describe any sensitivity analyses  Data were analyzed using STATA v14 (STATA Corp., College Station TX). Relative and absolute frequencies are reported for categorical variables, and mean and standard deviation are reported for quantitative variables. Follow-up time was calculated as the amount of time between January 1, 2016, or enrollment, which ever came later, and February 28, 2017. For those lost to follow-up, follow-up was calculated as one-half the amount of time between last contact with study personnel and the date recorded as lost to follow-up. A Poisson distribution was used to calculate 95% confidence intervals (CIs) for incidence rates. A binomial distribution was used to calculate 95% CIs for seroprevalence and proportion of cases among infections. We used generalized estimating equations assuming a Poisson distribution to calculate incidence rate ratios (IRR) for the risk of symptomatic ZIKV infection. Crude and adjusted IRRs were calculated using univariate and multivariate analysis, respectively. For analyses examining the risk of ZIKV infection and the risk of symptomatic ZIKV infection among all ZIKV infections, Poisson regression with robust standards errors was used. Analyses of the effect of DENV immunity were limited to children with known DENV infection histories. In addition, all ZIKV cases or infections that occurred prior to March 2016 (n=68) were excluded because: 1) The testing definition for Zika changed in February 2016, and 2) due to the potential DENV/ZIKV serological cross-reactivity in the ZIKV NS1 BOB assay when performed in samples collected early after the acute infection. Further, analyses of the effect of DENV immunity on Zika cases among ZIKV infections was limited to individuals who demonstrated a ZIKV NS1 BOB seroconversion regardless of case status. The variable age was explored as categorical and linear, and although the variable form did not impact the conclusions of the models, the continuous version generated a better model fit and thus was included in the final models. (Methods, paragraph 6) |
| Results | | |
| Participants | 13* | (a) Report numbers of individuals at each stage of study—eg numbers potentially eligible, examined for eligibility, confirmed eligible, included in the study, completing follow-up, and analysed |
|  |  | (b) Give reasons for non-participation at each stage |
|  |  | (c) Consider use of a flow diagram  A total of 3,893 children aged 2-14 years participated in the PDCS between January 1, 2016, and February 28, 2017. Of these, 3,053 (78.4%) participated throughout the entire study period, 386 (9.9%) were enrolled after January 1, 440 (11.3%) withdrew or were withdrawn from the study, and 14 (0.4%) both enrolled after January 1 and withdrew. A majority of the individuals who did not participate in the entire study period were 2-year old children who were enrolled after January 1 or children who turned 15 and aged out of the cohort. Other than the 15-year old subjects who aged out of the cohort, children who were withdrawn from the cohort or lost to follow-up were demographically similar to the cohort population. Participants were uniformly distributed by sex and year of age (Table 1). (Results, paragraph 1). |
| Descriptive data | 14* | (a) Give characteristics of study participants (eg demographic, clinical, social) and information on exposures and potential confounders  . |
|  |  | (b) Indicate number of participants with missing data for each variable of interest |
|  |  | (c) Summarise follow-up time (eg, average and total amount)  This information is summarized in Tables 1 & 2. |
| Outcome data | 15* | Report numbers of outcome events or summary measures over time  Table 2 |
| Main results | 16 | (*a*) Give unadjusted estimates and, if applicable, confounder-adjusted estimates and their precision (eg, 95% confidence interval). Make clear which confounders were adjusted for and why they were included |
|  |  | (*b*) Report category boundaries when continuous variables were categorized |
|  |  | (*c*) If relevant, consider translating estimates of relative risk into absolute risk for a meaningful time period  See Table 3. |
| Other analyses | 17 | Report other analyses done—eg analyses of subgroups and interactions, and sensitivity analyses  See Table 4. |
| Discussion | | |
| Key results | 18 | Summarise key results with reference to study objectives  We found a high incidence of both symptomatic and total ZIKV infections and estimated the effective reproduction number during the early growth phase of the epidemic to be 3.3-3.4. Girls and older children had a higher risk of ZIKV infection. Notably, PDCS children with prior documented DENV infection had a lower risk of developing symptoms when infected by ZIKV. (Discussion, paragraph 1) |
| Limitations | 19 | Discuss limitations of the study, taking into account sources of potential bias or imprecision. Discuss both direction and magnitude of any potential bias  Our study has several limitations; first, it is restricted to children and thus we cannot comment on the effect of prior DENV exposure in adults. Second, we are constrained by the epidemiology of our site, including the limited number of DENV infections in the years immediately preceding ZIKV introduction. Third, due to incomplete DENV exposure histories and/or the lack of paired annual serum samples, a subset of the cohort was excluded from the DENV immunity analyses. Fourth, for the identification of symptomatic ZIKV infections, we relied on participants to present at the Health Center, thus it is possible that we misclassified some infections as inapparent that were truly symptomatic. If presentation at the Health Center among children with symptomatic infections was related to DENV immunity, this could have biased our analyses. Fifth, determination of RT-PCR-negative symptomatic ZIKV infections and inapparent ZIKV infections relied on an algorithm that combines several ZIKV and DENV serological test results and on a ZIKV NS1 blockade-of-binding (BOB) ELISA, respectively. Although the algorithm was developed to maximize its diagnostic value and the ZIKV NS1 BOB assay has been characterized in different populations [33,34], we cannot exclude that several symptomatic and inapparent infections were misclassified. Sixth, risk of exposure to DENV and ZIKV are not independent, as the two viruses are both transmitted by the same mosquito and the underlying risk of exposure to arboviruses likely varies by home and school location as well as child. Underlying arboviral risk is a potential unmeasured confounder in our study. This may have biased our infection analysis towards the null, as children who had previously been exposed to DENV may be at higher risk for general arboviral infections than children who have not been exposed to DENV. In part to address this underlying relationship between DENV exposure risk and ZIKV exposure risk, we performed the analysis examining symptomatic ZIKV infections among ZIKV infections, which removes this potential source of bias. Importantly, the effect estimate for prior DENV immunity obtained in this analysis was similar to that obtained in the symptomatic ZIKV analysis, indicating that this potential source of bias did not significantly impact the symptomatic ZIKV analysis. (Discussion, paragraph 4) |
| Interpretation | 20 | Give a cautious overall interpretation of results considering objectives, limitations, multiplicity of analyses, results from similar studies, and other relevant evidence  Here, we show that children with a previous DENV infection have a lower risk of being symptomatic when infected by ZIKV after adjusting for sex and age, suggesting that previous DENV immunity may be protective against Zika. However, studies conducted using sera from humans exposed to DENV suggest that cross-reactive neutralizing responses induced by DENV against ZIKV are neither as strong nor as durable as those induced against heterologous DENV types [50-52]. Thus, we analyzed the risk of symptomatic Zika when infected in children with a recent (2015/2016 season) DENV infection. The IRR estimate was 0.57 (95% CI: 0.35, 0.92; p=0.020) when adjusted for age and sex; however, including prior DENV infection in the model increased the IRR estimate to 0.80 (95% CI: 0.47, 1.34; p=0.393). It is important to note that the incidence of DENV infection in the PDCS during the 2015/2016 season was low, which limits the power of the analysis. Additionally, cross-protection from a prior DENV infection might be provided through other antibody-mediated responses (e.g., antibody-dependent cellular cytotoxity) and/or through cross-protective CD8^+^ T cell responses [19, 53, 54]. (Discussion, paragraph 3) |
| Generalisability | 21 | Discuss the generalisability (external validity) of the study results  Our findings might be generalizable to other locations that have had DENV circulation in the years preceding ZIKV circulation, including a great majority of countries in the Americas. (Discussion, paragraph 5) |
| Other information | | |
| Funding | 22 | Give the source of funding and the role of the funders for the present study and, if applicable, for the original study on which the present article is based  This study was supported by grants R01 AI099631 (AB), P01 AI106695 (EH), and U19 AI118610 (EH) from the National Institute of Allergy and Infectious Diseases of the National Institutes of Health, as well as grant VE-1 (EH) from the Pediatric Dengue Vaccine Initiative of the Bill and Melinda Gates Foundation. The funders had no role in study design, data collection and analysis, decision to publish, or preparation of the manuscript. |

*Give information separately for exposed and unexposed groups.

**Note:** An Explanation and Elaboration article discusses each checklist item and gives methodological background and published examples of transparent reporting. The STROBE checklist is best used in conjunction with this article (freely available on the Web sites of PLoS Medicine at http://www.plosmedicine.org/, Annals of Internal Medicine at http://www.annals.org/, and Epidemiology at http://www.epidem.com/). Information on the STROBE Initiative is available at http://www.strobe-statement.org.
