# Supplementary material for: Prior dengue virus infection and risk of Zika: A pediatric cohort in Nicaragua
Source: PLoS Med. 2019 Jan 22;16(1):e1002726. doi: 10.1371/journal.pmed.1002726 (PMC6342296; doi:10.1371/journal.pmed.1002726)
Supplement: S1 Table — (DOCX) [file pmed.1002726.s003.docx]

**S1 Table. Characteristics of the participants included in the analysis of the effect of prior DENV exposure on the risk of symptomatic ZIKV infection.**

|  | **Full Cohort - n (%)** | | | | **Symptomatic ZIKV infection - n (%)** | | | | | **No symptomatic ZIKV infection - n (%)** | | | | |
| --- | --- | --- | --- | --- | --- | --- | --- | --- | --- | --- | --- | --- | --- | --- |
|  | **Prior DENV infection** * | | **Recent DENV infection** † | | **Prior DENV infection** * | | **Recent DENV infection** † | | | **Prior DENV infection** * | | | **Recent DENV infection** † | |
|  | **Yes** | **No** | **Yes** | **No** | **Yes** | **No** | **Yes** | **No** | **Yes** | | **No** | **Yes** | | **No** |
| By sex |  |  |  |  |  |  |  |  |  | |  |  | |  |
| Female | 358  (24.0) | 1132  (76.0) | 84  (5.6) | 1406  (94.4) | 46  (19.6) | 189  (80.4) | 6  (2.6) | 229  (97.5) | 312  (24.9) | | 943  (75.1) | 78  (6.2) | | 1177  (93.8) |
| Male | 385  (25.0) | 1152  (75.0) | 92  (6.0) | 1445  (94.0) | 38  (19.3) | 159  (80.7) | 9  (4.6) | 188  (95.4) | 347  (25.9) | | 993  (74.1) | 83  (6.2) | | 1257  (93.8) |
| By age (years) |  |  |  |  |  |  |  |  |  | |  |  | |  |
| 2-5 | 30  (3.6) | 795  (96.4) | 25  (3.0) | 800  (97.0) | 3  (3.4) | 86  (96.6) | 2  (2.2) | 87  (97.8) | 27  (3.7) | | 709  (96.3) | 23  (3.1) | | 713  (96.9) |
| 6-9 | 128  (13.0) | 855  (87.0) | 48  (4.9) | 935  (95.1) | 13  (8.7) | 137  (91.3) | 5  (3.3) | 145  (96.7) | 115  (13.8) | | 718  (86.2) | 43  (5.2) | | 790  (94.8) |
| 10-14 | 585  (48.0) | 634  (52.0) | 103  (8.4) | 1116  (91.6) | 68  (35.2) | 125  (64.8) | 8  (4.2) | 185  (95.8) | 517  (50.4) | | 509  (49.6) | 95  (9.3) | | 931  (90.7) |

* At least one inapparent or symptomatic DENV infection since the participant entered the PDCS until the 2015/2016 season.

† An inapparent or symptomatic infection during the 2015/2016 season.
