# Supplementary material for: Prior dengue virus infection and risk of Zika: A pediatric cohort in Nicaragua
Source: PLoS Med. 2019 Jan 22;16(1):e1002726. doi: 10.1371/journal.pmed.1002726 (PMC6342296; doi:10.1371/journal.pmed.1002726)
Supplement: S2 Table — (DOCX) [file pmed.1002726.s004.docx]

**S2 Table. Characteristics of the participants included in the analysis of the effect of prior DENV exposure on the risk of ZIKV infection and the risk of symptomatic ZIKV infection among those with ZIKV infection.**

|  | **Full Cohort - n (%)** | | | | **ZIKV infection - n (%)** | | | | | **No ZIKV infection - n (%)** | | | | |
| --- | --- | --- | --- | --- | --- | --- | --- | --- | --- | --- | --- | --- | --- | --- |
|  | **Prior DENV infection** * | | **Recent DENV infection** † | | **Prior DENV infection** * | | **Recent DENV infection** † | | | **Prior DENV infection** * | | | **Recent DENV infection** † | |
|  | **Yes** | **No** | **Yes** | **No** | **Yes** | **No** | **Yes** | **No** | **Yes** | | **No** | **Yes** | | **No** |
| By sex |  |  |  |  |  |  |  |  |  | |  |  | |  |
| Female | 282  (21.5) | 1031  (78.5) | 63  (4.8) | 1250  (95.2) | 128  (25.2) | 381  (74.9) | 22  (4.3) | 487  (95.7) | 154  (19.2) | | 650  (80.8) | 41  (5.1) | | 763  (94.9) |
| Male | 292  (21.7) | 1054  (78.3) | 71  (5.3) | 1275  (94.7) | 123  (27.2) | 329  (72.8) | 23  (5.1) | 429  (94.9) | 169  (18.9) | | 725  (81.1) | 48  (5.4) | | 846  (94.6) |
| By age (years) |  |  |  |  |  |  |  |  |  | |  |  | |  |
| 2-5 | 26  (3.5) | 709  (96.5) | 21  (2.9) | 714  (97.1) | 2  (1.1) | 173  (98.9) | 2  (1.1) | 173  (98.9) | 24  (4.3) | | 536  (95.7) | 19  (3.4) | | 541  (96.6) |
| 6-9 | 117  (12.4) | 829  (87.7) | 40  (4.2) | 906  (95.9) | 35  (11.1) | 280  (88.9) | 11  (3.5) | 304  (96.5) | 82  (13.0) | | 549  (87.0) | 29  (4.6) | | 602  (95.4) |
| 10-14 | 431  (44.1) | 547  (55.9) | 73  (7.5) | 905  (92.5) | 214  (45.4) | 257  (54.6) | 32  (6.8) | 439  (93.2) | 217  (42.8) | | 290  (57.2) | 41  (8.1) | | 466  (91.9) |

* At least one inapparent or symptomatic DENV infection since the participant entered the PDCS until the 2015/2016 season.

† An inapparent or symptomatic infection during the 2015/2016 season.
